# Early Life to Adult Brain Lipidome Dynamic: A Temporospatial Study Investigating Dietary Polar Lipid Supplementation Efficacy

**DOI:** 10.3389/fnut.2022.898655

**Published:** 2022-07-26

**Authors:** Manuel Oliveira, Kyoko Koshibu, Andreas Rytz, Francesca Giuffrida, Sebastien Sultan, Amaury Patin, Mathieu Gaudin, Aurore Tomezyk, Pascal Steiner, Nora Schneider

**Affiliations:** ^1^Brain Health Department, Nestlé Institute of Health Sciences, Nestlé Research, Société des Produits Nestlé S.A., Lausanne, Switzerland; ^2^Clinical Research Unit, Nestlé Research, Société des Produits Nestlé S.A., Lausanne, Switzerland; ^3^Analytical Science Department, Nestlé Institute of Analytical Sciences, Nestlé Research, Société des Produits Nestlé S.A., Lausanne, Switzerland; ^4^ImaBiotech SAS, Parc Eurasanté, Loos, France

**Keywords:** brain development, polar lipids, sphingolipids, phospholipids, MALDI-MSI

## Abstract

The lipid composition of the brain is well regulated during development, and the specific temporospatial distribution of various lipid species is essential for the development of optimal neural functions. Dietary lipids are the main source of brain lipids and thus contribute to the brain lipidome. Human milk is the only source of a dietary lipids for exclusively breastfed infant. Notably, it contains milk fat globule membrane (MFGM) enriched in polar lipids (PL). While early life is a key for early brain development, the interplay between dietary intake of polar lipids and spatial dynamics of lipid distribution during brain development is poorly understood. Here, we carried out an exploratory study to assess the early postnatal temporal profiling of brain lipidome between postnatal day (PND) 7 and PND 50 using matrix-assisted laser desorption ionization as a mass spectrometry imaging (MALDI-MSI) in an *in vivo* preclinical model. We also assessed the effect of chronic supplementation with PL extracted from alpha-lactalbumin-enriched whey protein concentrate (WPC) containing 10% lipids, including major lipid classes found in the brain (37% phospholipids and 15% sphingomyelin). MALDI-MSI of the spatial and temporal accretion of lipid species during brain development showed that the brain lipidome is changing heterogeneously along time during brain development. In addition, increases in 400+ PL supplement-dependent lipids were observed. PL supplementation had significant spatial and temporal effect on specific fatty esters, glycerophosphocholines, glycerophosphoethanolamines, and phosphosphingolipids. Interestingly, the average levels of these lipids per brain area tended to be constant in various brain structures across the age groups, paralleling the general brain growth. In contrast, other lipids, such as cytidine diphosphate diacylglycerol, diacylglycerophosphates, phosphocholines, specific ether-phosphoethanolamines, phosphosphingolipids, glycerophosphoinositols, and glycerophosphoserines showed clear age-dependent changes uncoupled from the general brain growth. These results suggest that the dietary PL supplementation may preferentially provide the building blocks for the general brain growth during development. Our findings add to the understanding of brain-nutrient relations, their temporospatial dynamics, and potential impact on neurodevelopment.

## Introduction

The brain contains the highest proportion of the total lipid content found in the human body with the exception of adipose tissue (1). The major classes of lipids found in a human brain throughout life are sphingolipids, glycerophospholipids, and cholesterols (2, 3). These lipids form the essential building blocks for the cellular membranes of neural cells that are critical for establishing proper brain networks through, for example, myelination, synaptogenesis, and synaptic transmissions, to support healthy brain development (4, 5). The lipid content in the brain rapidly increases after birth and remains highly dynamic throughout the lifespan with a specific spatial distribution in mammals (6–9). The major phospholipids found in mammalian brains are phosphatidylethanolamine (PE) and phosphatidylcholine (PC), followed by phosphatidylserine (PS), phosphatidylinositol (PI), and sphingomyelin (SM) (10, 11). Phospholipids and sphingolipids are the types of polar lipids that have been shown to play critical roles in several physiological mechanisms, including neural maturation, myelination, neuronal functions, and cell signaling during cognitive development (12). Moreover, these phospholipids show brain region-specific distributions (13); in brain regions critical for learning and decision-making, memory formation and emotional processing, such as prefrontal cortex, hippocampus, and amygdala, very high levels of PC and PE have been detected in adult rats, suggesting that region-specific lipid composition contributes to healthy brain functions (14–17). Furthermore, alteration in the brain lipidome and lipid metabolism has been observed in neurodevelopmental disorders, such as autism and attention deficit/hyperactivity disorder reinforcing the idea that they play a central role for optimal brain function and development (18, 19).

Recently, studies have demonstrated substantial impact of diet and nutritional interventions on the brain lipid composition (20, 21). Maliković et al. (20) demonstrated that energy reduced diet during adulthood can modulate the brain lipid levels, which resulted in impaired reversal learning. In another study, feeding rats with cereal modified brain cholesterol, vitamin E, and fatty acid distributions (22). Furthermore, early life deficits in essential nutrients, such as lipids, vitamins, and minerals, are associated with impairments in human brain connectivity and cognitive development, reinforcing the notion that proper early life nutrition is essential for healthy brain development (23, 24). In line with the evidence in humans, several preclinical studies manipulating dietary or peripheral lipid levels have reported changes in the brain lipidome that were associated with behavioral changes, such as anxiety-like behaviors and learning defects, for example (25–28). These findings support the notion that early life nutrition plays a critical role in ensuring the appropriate cerebral abundance of specific lipid species.

Early life nutrition with adequate levels of lipids and lipid sources are critical for healthy brain development. As such, breast milk, which is recognized as the optimal source of nutrition for infants, contains key nutritional and non-nutritional components essential for infant development and health, including lipids (29, 30). In mammals, breast milk lipids promote, for example, physical growth, neurodevelopment, and maturation of the immune system by providing energy and relevant cellular membrane building blocks, such as triglycerides, cholesterols, free fatty acids, and polar lipids (31–33). In breast milk, lipids are released in lipid droplets called milk fat globules, containing non-polar lipids surrounded by a biological membrane, named milk fat globule membrane (MFGM), containing polar lipids, glycolipids, and proteins (34, 35). In mature human milk, polar lipids, such as glycerophospholipids and sphingomyelin account for 0.2–1.0% of the 3.4 g/100 ml milk fat found at 99% in the form of triglyceride (32, 36). In fact, preclinical studies have assessed the effect of early MFGM supplementation on brain lipids and cognitive functions (37, 38). For example, Mudd et al. have shown that the treatment of piglets with MFGM, lactoferrin, and prebiotics from PND 2 to PND 30 induced improvements in T-maze memory task and redistribution of brain lipidome at the end of the intervention (37). Furthermore, Sultan et al. have recently reported that the polar lipids, which are the components of MFGM, can induce an improvement in a Morris water maze spatial memory task in rats when they are treated from PND 7 to PND 72 (38). These studies highlight the importance of lipid supplementation for brain function.

While early life nutrition and the importance of lipids in early brain development have been established, there exist only a few studies reporting the spatiotemporal changes in specific lipid species during the brain development. Moreover, it is currently unknown whether lipid supplementation can modulate brain lipidome in a specific spatiotemporal sequence and how such a pattern may be crucial to support brain development. We therefore investigated in an exploratory study the effect of dietary polar lipid supplementation on lipid diversity and distribution during brain development, from early life to adulthood in a preclinical model. Polar lipids were chosen as a choice of supplement, because they are important natural constituents in human breast milk and are the building blocks of cell membrane (39). Polar lipid fraction contained in a uniquely processed whey protein concentrate enriched in alpha-lactalbumin and phospholipids, including sphingomyelin was used as dietary supplement (40). A cutting edge *in situ* imaging technology, matrix-assisted laser desorption ionization as a mass spectrometry imaging (MALDI-MSI), was used to identify lipid compositional changes in the brain in response to the PL supplementation and then to map their spatial distribution within 13 brain regions, along different developmental stages between PND 7 and PND 50 (41–43). MALDI-MSI is a valuable tool to study the spatial distributions of lipids with a high level of sensitivity (44). Using this approach, we were able to precisely profile brain lipidome dynamic and identify specific spatiotemporal signatures of molecules belonging to different lipid classes, influenced by PL supplementation. Our data suggest that nutrition strategy could be implemented to influence early life brain development through modulation of lipid biosynthesis pathways.

## Materials and Methods

### Animals

All animal experimentations were conducted by Amylgen (Montferrier-sur-Lez, France). The experimental procedure involving animals was reviewed by ethics committees and conducted in strict adherence to the European Union directive of September 22, 2010 (2010/63/UE). Amylgen's authorization was delivered by the Direction Régionale de l'Alimentation, de l'Agriculture et de la Forêt du Languedoc-Roussillon - France (agreement #A 34-169-002; 02 May 2014). In brief, pregnant Wistar rats with E14 embryos were purchased from JANVIER (Saint Berthevin, France) and were kept under a 12-h/12-h light/dark cycle (lights off at 07:00 pm) at room temperature (21–22°C) and humidity (40–60%)-controlled environment with *ad libitum* food and water. Within 48 h after birth, 4 litters were adjusted to eight males based on the body weight and fostered to recipient lactating dams.

### Polar Lipid Supplementation

**Figure 1:**
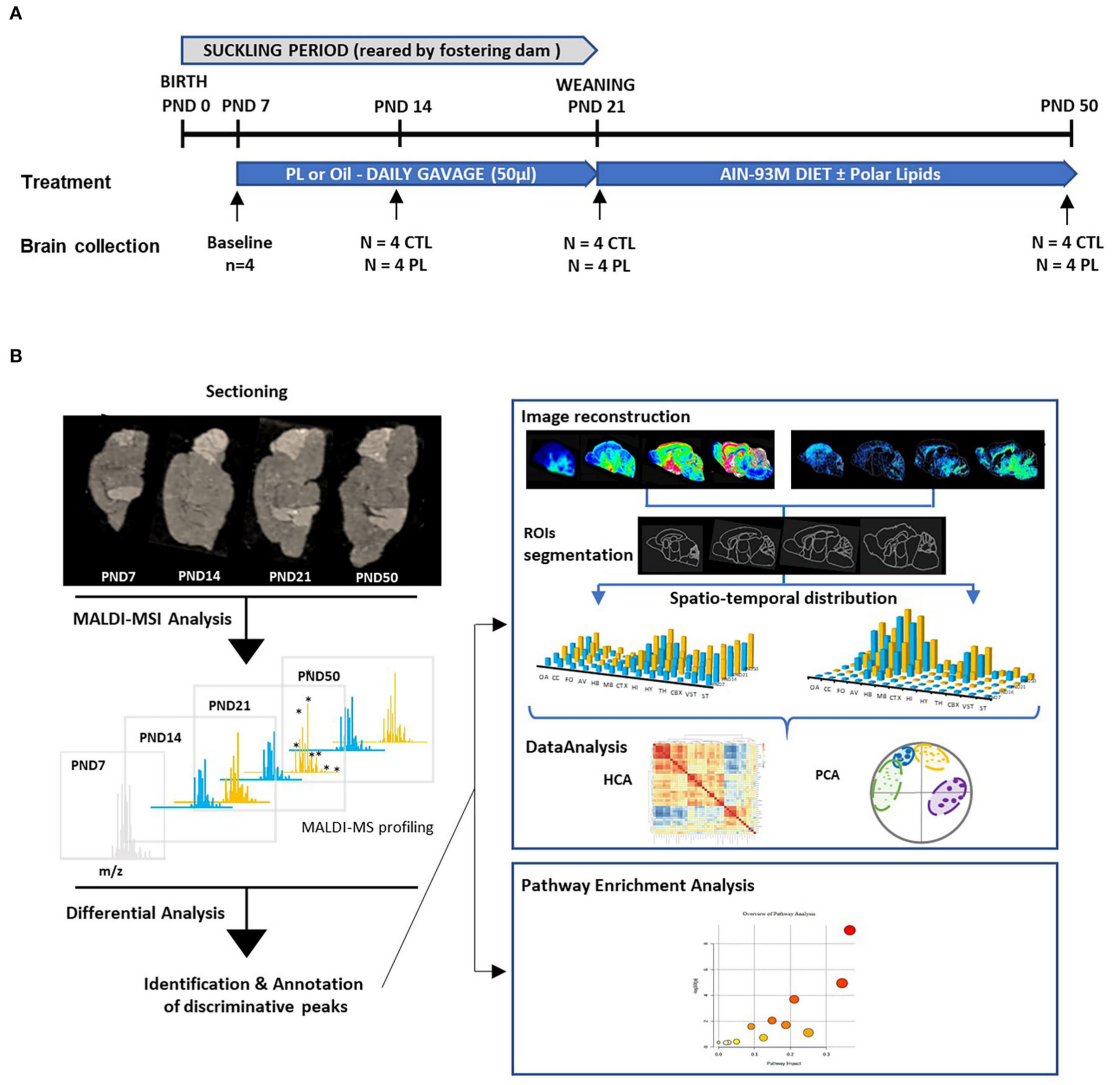
Experimental design. **(A)** The experimental paradigm and the number of animals terminated at each time point are shown. At birth, 8 male rat pups were randomly allocated into 4 different litters reared by lactating dams. At PND 7, pups within each litter were randomly assigned to control (CTL) and polar lipids (PL) (*n* = 4 per group). Nutritional supplementation started at PND 7 and lasted until the animals were terminated at respective time points. About 1 Litter, consisting of 4 control and 4 PL animals, was terminated at PND 7, 14, 21 and 50, and the brains were collected. **(B)** A schematic overview of the MALDI-MSI analysis is described. In brief, 10-μm-thick sagittal sections of brains from different time points were mounted on conductive MALDI slides. A full scan MALDI-MSI spectra was collected in positive and negative ionization mode. Discriminative peaks were identified by differential analysis between the two groups. Pathway enrichment analysis was performed using annotated molecular species. To obtain their spatiotemporal distribution, reconstituted molecular images were segmented in ROIs based on histological staining performed in juxtaposed section. Multivariate analysis was performed to identify pattern similarities among different annotated species.

At postnatal day (PND) 7, pups from each litter were randomly allocated to either the polar lipid-supplemented or control group (*n* = 4 male pups per group in each litter) and either received a daily supplement of 3.3 mg/10 μl/g body weight of PL extract emulsion suspended in water using 22-gauge feeding tubes (product number FTP 22-25; Instech Laboratories, Plymouth, PA, USA) or oil, respectively, until PND 21 (Figure 1A). To ensure balanced energy intake, control animals received a blend of oil containing 35% corn oil (Sofinol S.A., Manno, Switzerland), 50% soybean oil (Sofinol S.A., Manno, Switzerland), and 15% cocoa butter (Gerkens Cacao®, EJ Deventer, The Netherlands). The PL supplement was warmed at 52°C and emulsified (33% w/w) by vigorous shaking in sterile water before supplementation, whereas the control lipid blend was administered as such. Both supplementations were done on the top of dam's milk. At PND21, weaned rats were housed in pairs and polar lipid extract was incorporated at 1.3% in the AIN93G semi-purified diets from PND 21 to PND 40 and AIN93M from PND 41 to PND 50 as a part of the fat source (Supplementary Table 1). Body weight and food intake were monitored two times a week throughout the study and were not significantly different among the groups (Supplementary Figure 1). At PND 7, 14, 21, and 50, whole brains were quickly removed and immediately frozen in dry-ice chilled isopentane (Sigma Aldrich chimie, St Quentin Fallavier, France) for 3 min and then wrapped in a piece of aluminum foil and stored at −80°C.

### Polar Lipid Extract

Polar lipids (not intended for human consumption) were extracted from a single batch of alpha-lactalbumin-enriched WPC containing 10% lipids (40) using a conventional Folch method (45). Briefly, whey proteins were mixed at a sample-to-extraction solvent ratio 1:5 (w/v) in chloroform/methanol solution (2:1, v/v) (Sigma Aldrich chimie, St Quentin Fallavier, France) and heated at 50°C under mechanical stirring. Mixture was passed through a 5-μm sintered glass filter cartridge to guarantee a maximal yield, and solvents from the liquid phase were removed by vacuum evaporation. The composition of the resulting polar lipid extract was determined with an untargeted approach (46): 42.7% of phospholipids, 55.6% of triacylglycerol, 0.6% of lyso-phosphatidylcholine, 0.9% of lyso-phosphoethanolamine, 0.2% plasmalogen-phosphatidylethanolamine. Phospholipid species were analyzed by LC/MS-MS (47) and contained the following MFGM components: 14.6 g sphingomyelin (SM), 14.5 g phosphatidylcholine (PC), 1.1 g phosphatidylserine (PS), 5.5 g phosphatidylethanolamine (PE), and 1.1 g phosphatidylinositol (PI) in 100 g of total extract. Profiling of phospholipid molecular species was determined for SM, PE, PC, and PI (Supplementary Table 2).

### Tissue Preparation

MALDI-MSI, histological, and immunohistochemical analyses were conducted by ImaBiotech (Lille, France). The left hemisphere was sectioned at 10 μm thickness with a HM 360 cryostat at −24°C (Thermo Fisher Scientific, Walldorf, Germany). A total of four consecutive sagittal sections were collected with a target plane defined as 1.13 mm (lateral) according to the Paxinos and Watson Brain Atlas. The first and third sections were thaw-mounted on indium tin oxide (ITO)-coated glass slides (Delta Technologies, Loveland, CO, USA) for the MALDI-MSI analysis, the second section was thaw-mounted on SuperFrost Plus™ slides (VWR, Fontenay-sous-Bois, France) for the Nissl staining, and the fourth section was thaw-mounted on SuperFrost Plus™ slides for synaptophysin immunohistological staining. A section of a drug-spiked liver homogenate was mounted on each MALDI-MSI slide as a quality control to monitor inter-slide variability.

### Histological and Immunohistological Staining

To study the spatial distribution of molecular species in distinct neuroanatomical structures, superimposition of coregistered high-resolution scan of MALDI MSI with Nissl staining and synaptophysin immunohistochemistry on juxtaposed brain tissue sections was used to segment regions of interest (ROIs). The tissue sections collected on the SuperFrost Plus™ slides were dried and fixed in 4% paraformaldehyde (PFA) in phosphate-buffered saline (PBS), pH7.4 (Electron microscopy science, Hatfield, USA) for 10 min at room temperature. The sections were then blocked in 0.3% H_2_O_2_ in PBS for 10 min and washed with PBS for 10 min before being incubated in 1% normal goat serum in PBS with 0.1% Triton (Sigma Aldrich chimie, St Quentin Fallavier, France) for 1 h. After blocking, sections were incubated with mouse monoclonal anti-synaptophysin (Abcam, Ab8049) diluted to 1:10 in PBS containing 1% normal goat serum and 0.1% Triton overnight at 4°C. Sections were then washed three times with PBS (10 min per wash) and incubated in a biotinylated secondary anti-mouse antibody for 30 min at room temperature. The biotin signal was visualized using an immunohistochemistry HRP/DAB detection kit (Mouse and Rabbit Specific HRP/DAB IHC Detection Kit-Micro-polymer-ab236466, Abcam, Cambridge, UK), following the supplier's protocol. The digital images were captured using a digital slide scanner (3D Histech Pannoramic, Budapest, Hungary) and a confocal microscope (Fv10i, Fluoview; Olympus, Rungis, France) and analyzed using a FV10-ASW 2.1 viewer (Olympus, Rungis, France) and Image Scope v12.1.0.5029 (Leica Biosystems, Buccinasco MI, Italy).

### MALDI-MSI

Brain sections adjacent to those used for the histological and immunohistological staining were sprayed with a matrix using an automatic TM-Sprayer (HTX-Imaging, Chapel Hill, NC, USA). The matrix was composed of 40 mg/ml of 2,5-DHB in methanol/water 1:1 (v:v) + 0.1% trifluoroacetic acid (TFA), and 10 mg/ml of 1,5-DAN in acetonitrile/water 1:1 (v:v) used for the analysis in positive and negative ion modes, respectively (48). MALDI-MSI analyses were conducted by using Solarix FTICR-MS (Bruker Daltonics, Bremen, Germany) within a mass-to-charge ratio (m/z) range of 50–2,000 Th in both positive and negative ionization modes. Ionization was performed in a MALDI mode using a SmartBeam II laser operated at a repetition rate of 2,000 Hz. The mass spectrum obtained for each position of the images corresponded to the average of 300 consecutive laser shots on the same location. Prior to each data acquisition, an external calibration was performed using well-known endogenous compounds and MALDI matrix ions. Brain MS images were acquired at a spatial resolution of 125 μm. Mass spectra were acquired with ftmsControl 2.0 and FlexImaging 4.1 (Bruker Daltonics, Billerica, MA, USA) and visualized by DataAnalysis v4.1 (Bruker Daltonics, Billerica, MA, USA). Processing and analysis of the MALDI-MSI datasets was performed by Multimaging™ 1.1 (ImaBiotech, Loos, France). Intensity scales were built by synchronizing all images and normalizing intensities to the pixel of maximum intensity across the entire dataset. The average intensity for each m/z was calculated for the entire brain and in specific regions of interest (ROI) as the average intensity of every pixel. The ROIs were manually delineated in 13 brain regions including olfactory area (OA), ventral striatum (vST), striatum (ST), cerebral cortex (CTX), hippocampus (HIP), hypothalamus (HYP), thalamus (TH), midbrain (MB), hindbrain (HB), cerebellar cortex (CBX), arbor vitae (AV), fornix (FO), corpus callosum (CC), and whole tissue section according to the rat atlas (Supplementary Figure 2) (49–51).

### Peak Annotation

The hit lipid molecular species were assigned using the Metlin (52, 53) and LipidMaps public databases (54) with the mass accuracy as a first criterion with a wide tolerance (10 ppm) to review a large list of proposed annotations. A knowledge-based annotation strategy was then followed (55). Filtering of the proposed annotation was carried out by examining the isotopic pattern, the consistency of the adduct formed with the chemical structure of the molecule and number of carbons in the fatty chains. Without using MS/MS, chemical variants with the same number of acyl chains and double bonds could not be discriminated.

### Annotation of Lipid Classes and Species

Nomenclature described by Fahy et al. (56) was used. Using this naming system, three categories were identified: fatty acyls, glycerophospholipids, and sphingolipids. Fatty acyls included fatty acids (FA), fatty acyl carnitines (CAR), and wax monoesters (WE). Glycerophospholipids included phosphatidic acid (PA), CDP-diacylglycerol (CDP-DG), phosphatidylcholine (PC), phosphatidylethanolamine (PE), phosphatidylglycerol (PG), Phosphatidylinositol (PI), phosphatidylserine (PS), alkyl- or alkenyl-phosphatidylethanolamine (PE-O / PE-P), alkyl- or alkenyl- phosphatidylcholine (PC-O / PC-P), alkyl- or alkenyl- phosphatidylserine (PS-O / PS-P), lysophosphatidic acid (LPA), and lysophosphatidylcholine (LPC). Sphingolipids included sulfatide (ST), hexosylceramide (Hex), fast-migrating cerebroside (FMC), phosphatidylethanolamine-ceramide (PE-Cer), and sphingomyelin (SM). The lipid species were annotated according to their molecular composition as follows: [lipid class] - [(sum of carbons atoms): (sum of double bonds)].

### Enrichment Analysis

Pathways were assigned according to the KEGG database annotation using MetaboAnalyst 5.0 (57). Over-representation of lipid classes and pathways compared to random sampling from detected lipids with annotations were tested using one-tailed Fisher's exact tests and hypergeometric tests, respectively, followed by Holm–Bonferroni (BH) corrections. Significant enrichment was defined as BH-corrected *p* < 0.05.

### MALDI-MSI Data Analysis

The MALDI-MSI data featured, respectively, 3,108 features in positive and 2,133 in negative ionization modes with 4 animals per Treatment x Development condition (i.e., PND 7, 14, 21, and 50 days for both CTL and PL). Individual data were (1) log-transformed to account for log-normal distribution by peak, (2) standardized to z-scores to give same weight to all peaks independently of abundance and (3) 5% winsorized to minimize the influence of individual outliers (58). Principal component analysis (PCA) of these data was performed to simultaneously visualize the correlation structure of features (biplot loadings) and the multivariate discrimination between conditions (biplot scores, average of 4 animals) (59). Generalized linear models (GLMs) were used to estimate the contribution of treatment and development, as well as their interaction on the signal. While at least one main effect was highly significant (*p* < 0.05) for all peaks, the interaction was systematically non-significant (*p* > 0.05). Features for which PL induced significantly higher intensities than CTL (*p* < 0.05) were grouped by the deciles of increasing m/z. In each decile, 60% smallest *p*-value were considered, to select peaks representing the whole m/z spectra. Among these selected peaks (respectively, 193 in positive and 281 in negative ionization modes), only 39 could be annotated. For those annotated peaks, MALDI signal intensity was further analyzed for 13 regions of interest (ROIs). These enhanced data were analyzed using the same techniques, namely, PCA biplot and GLM model (with ROI as additional parameter). These 39 species were further clustered using hierarchical clustering analysis (HCA) and visualized using Pearson correlation heatmap (60) (Figure 1B).

## Results

### Age-Dependent Lipid Distribution During Brain Development Using MALDI-MSI

**Figure 2:**
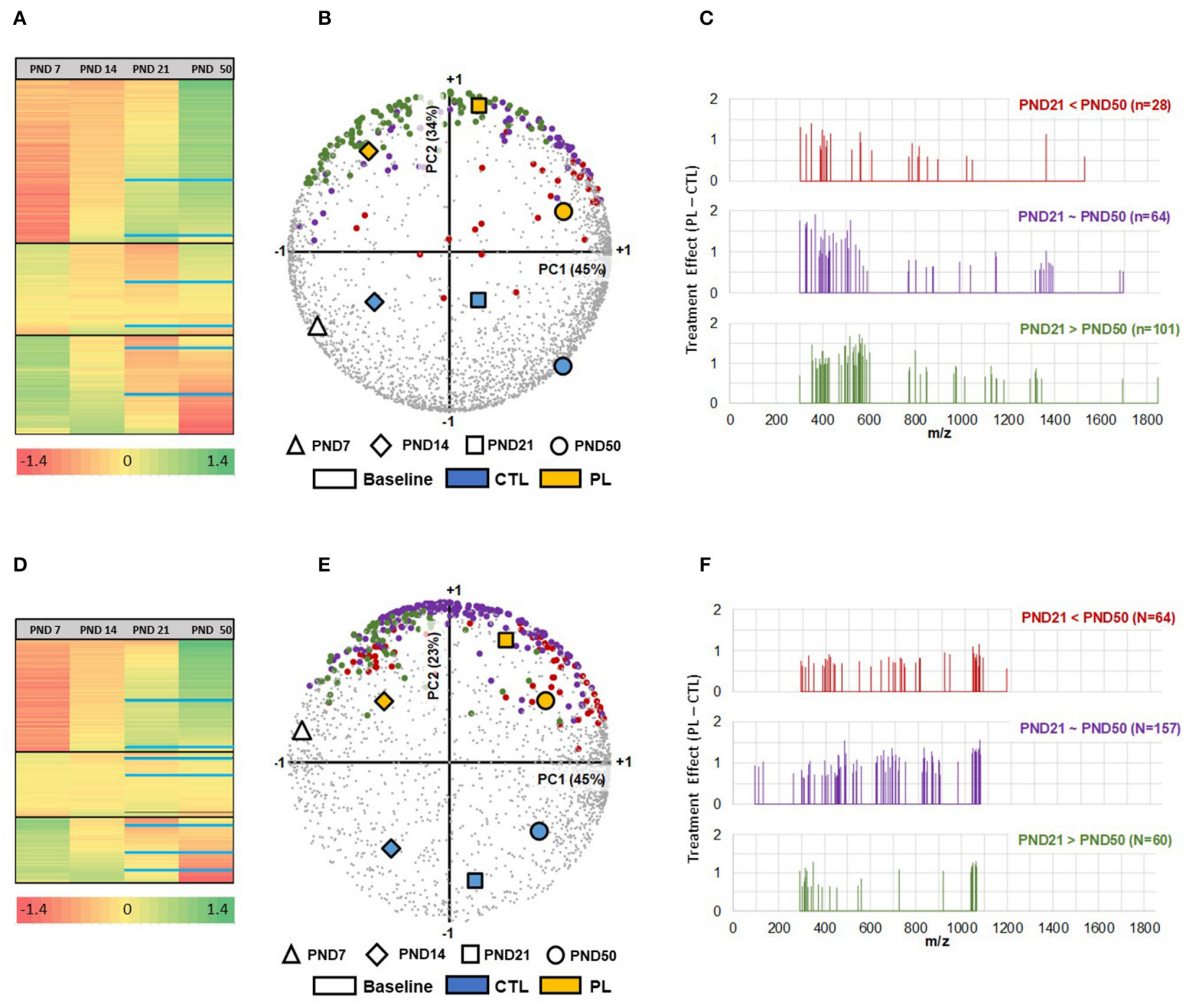
Neurodevelopmental changes in brain lipidome and the impact of the PL supplementation. **(A,D)** In total, three developmental clusters (increasing, no change, and decreasing with age) of brain lipidome were detected using HCA, shown for positive and negative ionization mode, respectively. The black solid lines divide the three clusters within the HCA heatmap. Within each cluster, 3 additional subclusters were identified, divided by blue solid lines within each cluster, for PND 21 and PND 50. PCA Biplot of first and second principal components with Treatment x Age positioned for m/z detected in **(B)** positive ionization mode (*n* = 3,108) and **(E)** negative ionization mode (*n* = 2,133). PC1 (plotted on x-axis), accounting for 45% in both modes, is highly associated with developmental age, whereas PC2 (plotted on y-axis), accounting for 34% in positive and 23% in negative modes, respectively, is strongly differentiating the two treatments, revealing that more than 70% of total variability is explained by the independent contributions of age and treatment effects. Selected m/z are further colored accordingly to their evolution pattern between PND 21 and PND 50 **(C,F)**. The m/z identified by differential analysis at PND 21 in positive and negative ionization modes were subcategorized into molecular species that increased (red), decreased (green) and stayed the same (purple) at PND 50 compared to PND 21. The selected hits 193 (+) and 281 (-), are shown as a function of m/z. The x-axis corresponds to the mass range, whereas the y-axis shows the peak intensity difference between experimental groups expressed in log. All analysis were performed on z-scores of log-transformed peak intensities.

MALDI-MSI technology was used to identify and visualize the temporal and spatial changes in the distribution of lipid species and potential response to dietary PL supplementation in growing rat brains. An untargeted approach in a full scan mode (50 and 2,000 Th) allowed the coverage of 3,108 and 2,133 features in positive and negative ionization modes, respectively. Their respective variations over a m/z range of 300 to 1,900 and 93 to 1,870 highlighted the chemical diversity of molecules underlying the complexity of brain lipidome at each developmental time point. Multivariate analysis using HCA and PCA biplot was carried out to visualize the relationship between groups and features detected in both ionization modes (Figure 2). In general, three major clusters were identified using HCA with one cluster of features increasing, while others either staying approximately at the same level or decreasing between PND 7 to PND 50 (Figures 2A,D). Additional subclusters were observed within each cluster, demonstrating the complexity of lipidome development. Approximately 26% of features were the highest at PND 7 and decreased over time. In contrast, the levels of approximately 46% of features gradually increased and peaked at PND 50. The rest stayed approximately the same. More specifically, the age-dependent changes in the features distribution were most prominent after PND 14 as depicted by the greater separation of the groups at PND 21 and 50 along the PC1 axis in the PCA (Figures 2B,E). Furthermore, the age-associated lipidome trajectory significantly shifted after PND 21 in both ionization modes, indicating a shift in the lipid distribution along time. Thus, the different groups were clearly segregated by age along the PC1 axis of the PCA, covering 45% of the variability in both ionization modes, indicating a temporal divergence in the composition of the lipidome at different ages. These results show that the brain lipidome is changing heterogeneously along time during brain development.

### Effect of Lipid Supplementation on Whole Brain Lipid Composition During Development

The PL-supplemented and control groups were clearly separated along the PC2 axis of the PCA, regardless of the age group, which accounted for 34 and 23% of the variance of the data acquired in positive and negative modes, respectively. Using this method, we found that subsets of features showed a clear response to the PL supplementation at all developmental time points, when the signal intensity was averaged over the entire brain region (Figures 2B,E). The effect of PL supplementation was prominently observed at PND 21 and the ion peaks that were significantly increased by the PL supplementation at this time point were annotated. The result of dataset reduction highlighted that the effect of the supplementation was stronger in the lipid species with a relative abundance peaking before PND 21 (PND 21 > PND 50 and PND 21 = PND 50). A reduction in molecular ion peaks was observed after PND 21 (Figures 2C,F). Moreover, the number of molecular ion species annotated for these categories of lipids was also greater than the molecular ions peaking at PND 50 (PND 21 < PND 50: 28 & 64 species; PND 21 = PND 50: 64 & 157 species; PND 21 > PND 50: 101 & 60 species, respectively, for positive and negative ionization modes). These results suggest a time-dependent malleability of brain lipidome to exogeneous supplementation.

Among all the different ions that were modulated by PL supplementation (193 from the positive mode and 281 from the negative mode), 39 of them were annotated (Supplementary Table 3). These lipids belonged to three main lipid categories: fatty acyls (4), glycerophospholipids including plasmalogens (ether-linked glycerophospholipids, 28) and sphingolipids (9). The fatty acyls included two classes of molecules, fatty acid [FA (22:6)] and acyl-carnitine [CAR (16:0), CAR (16:1), and CAR (16:2)]. For the glycerophospholipids category, the diacyl, alkenyl-acyl (P-) also known as plasmalogen and alkyl-acyl (O-) forms of molecules belonging to 7 different classes were annotated. The classes under this category included phosphatidic acids [PA (38:5) and PA (38:3)], cytidine diphosphate-diacylglycerol [CDP-DG (40:7)], lysophospholipids [LPA (22:6), and LPC(O-14:1)], phosphatidylcholines [PC(35:3), PC(42:8), and PC(O-36:1)|PC(P-36:0)], phosphatidylethanolamines [PE(36:2), PE(36:4), PE(42:5), PE(P-42:4) PE(O-42:6), PE(O-36:5)|PE(P-36:4), PE(O-40:4)|PE(P-40:3), PE(O-40:1)|PE(P-40:0), PE(O-36:3)|PE(P-36:2) and PE(O-40:2)|PE(P-40:1)], phosphatidylserines [PS(41:5), PS(O-36:2)|PS(P-36:1), PS(O-36-3) and PS(P-36:2)], and phosphatidylinositols [PI(34:0), PI(36:3), PI(38:4), PI(41:0). Finally, there were 3 classes of lipids annotated for the sphingolipid category including sphingomyelins (SM) or its analog ceramide phosphoethanolamine as PE-Cer (t40:1), PE-Cer (d38:2)|SM (d36:2) PE-Cer (d38:1)|SM (d36:1), PE-Cer (d36:1)|SM (d34:1)]. Some glycosphingolipids, such as sulfatide (ST), hexosylceramide (HexCer), and fast-migrating cerebrosides (FMC), were also annotated as follows: ST(d18:1/24:1), HexCer(d34:1), and FMC-6(d18:1/22:0(2-OH). The putative annotation were performed only on statistically significant features. Therefore, it may explain why the adducts observed for a given lipid class may differ from one lipid to another, depending on the variability associated with adduct formation. Some odd carbon number chains were annotated, whereas even acyl/alkyl chains were supposed to be more frequent. When alternative annotations were possible, even chain lengths were favored. When not, these putative annotations were discarded. As these fatty acyl chains cannot come from rat biosynthesis, we may hypothesize that they come from dietary origin (odd chain free fatty acids) and subsequent incorporation during phospholipid biosynthesis.

**Figure 3:**
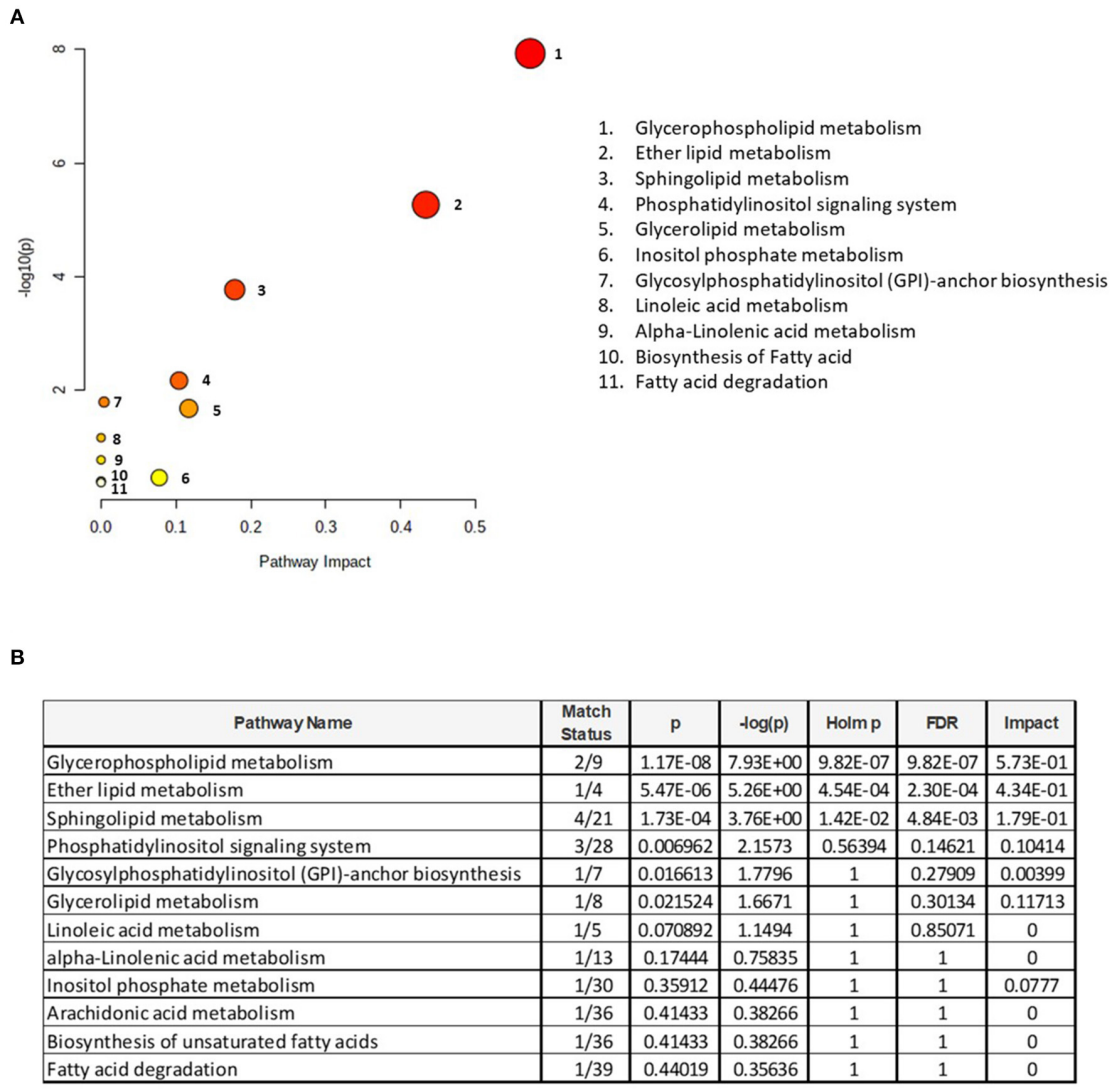
Pathway enrichment analysis. **(A)** Identification of metabolomic pathways altered by the supplementation and **(B)** metabolome view with all matched pathways according to the *p*-values from the pathway enrichment analysis and pathway impact values from the pathway topology analysis are presented. The size of the circle reflects the impact on the pathway, where larger circles denote bigger impacts. The color intensities increase with reducing *p*-value. Match status is the total number of annotated lipids in the pathway. The raw p is the original *p*-values calculated from the enrichment analysis. The holm p is the value adjusted by Holm–Bonferroni method. The impact is the pathway impact value calculated from the pathway topology analysis.

To understand the metabolic pathways impacted by the PL supplementation, a pathway enrichment analysis was conducted for these 39 lipids. A total of 12 metabolic pathways were identified for this whole brain analysis (Figure 3A). The top 3 metabolic pathways were statistically significant (FDR < 0.05) and included (1) glycerophospholipid metabolism, (2) ether lipid metabolism, and (3) sphingolipid metabolism (Figure 3B).

### Brain Spatiotemporal Distribution of PL Supplement-Sensitive Lipid Species

**Figure 4:**
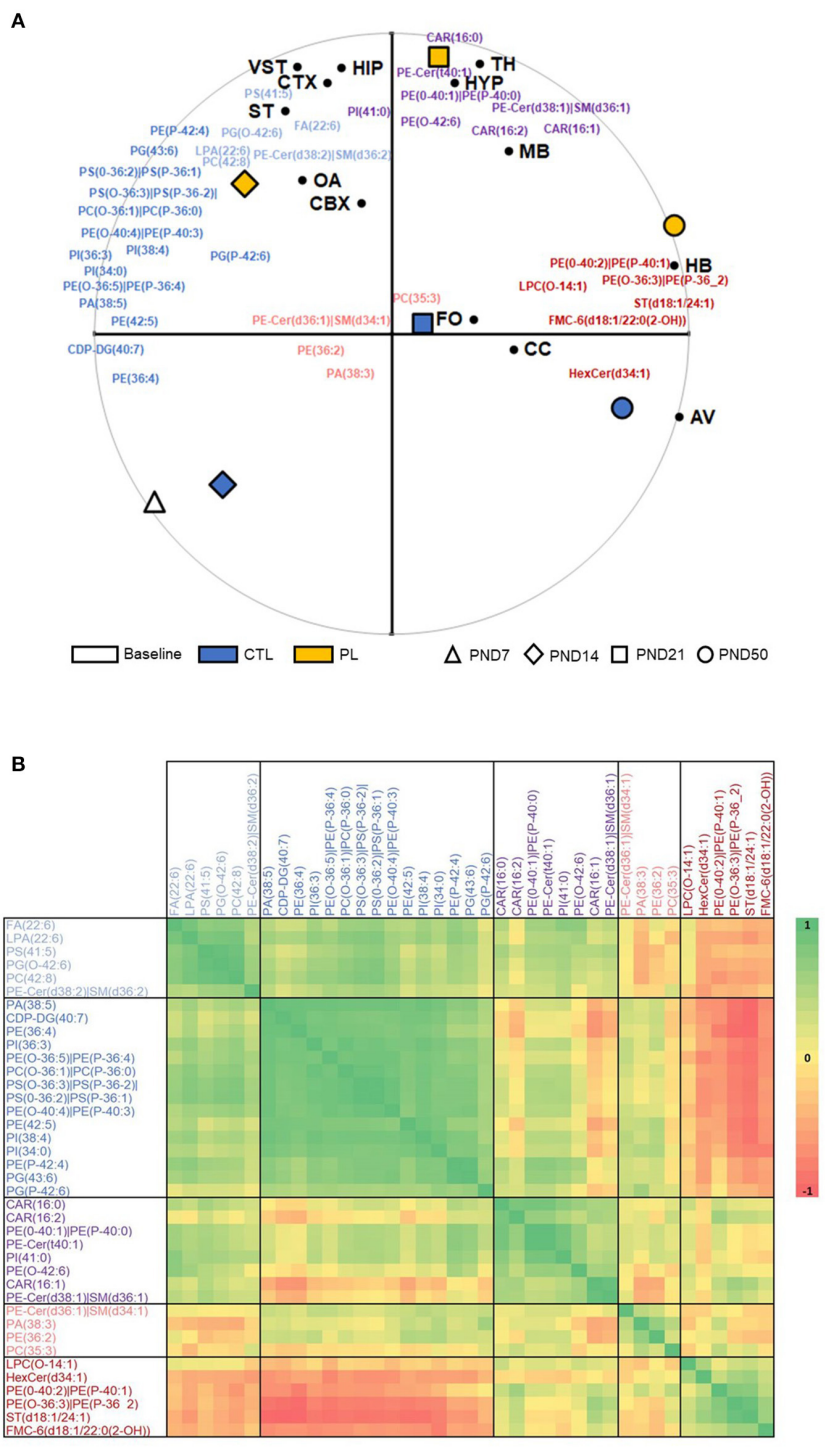
Time-coordinated spatial distribution of annotated lipids. **(A)** Biplot of first and second principal components with Treatment x Age positioned relative to all annotated species are shown. PC1, accounting for 40%, is highly associated with developmental age, whereas PC2, accounting for 26%, is strongly differentiating the two treatments, revealing a similar pattern for these 39 annotated species compared to all 5241 m/z detected in the two ionization modes. The average positions of ROIs are further superimposed on this biplot. **(B)** Pearson correlation heatmap of all annotated species based on Treatment x Age x ROI z-scores on log-transformed data is shown. A total of five clusters were identified using the furthest neighbor aggregation hierarchical clustering. OA, olfactory area; CTX, cortex; ST, striatum; vST, ventral striatum; TH, thalamus; HYP, hypothalamus; MB, midbrain; HB, hindbrain; CBX, cerebellum; AV, arbor vitae; CC, corpus callosum; FO, fornix.

To investigate the developmental changes and temporal specificity of the PL supplementation in specific brain structures, the MALDI signal intensity for the 39 annotated lipids was analyzed for 13 ROIs at all time points (Supplementary Figure 2). The PCA biplot and HCA were carried out on the intensity of the peak spectra collected within the ROIs (Figure 4). In this plot, the effect of PL on the overall lipid composition is indicated as a clear separation of the orange diamond, square, and circle, representing the overall PL effect at each time point, from the blue diamond, square, and circle, representing the control levels, along the PC2 axis (Figure 4A). HCA regrouped lipids in five clusters sharing similar spatiotemporal patterns (Figure 4B).

#### Fatty Acyls

##### Fatty Acids

**Figure 5:**
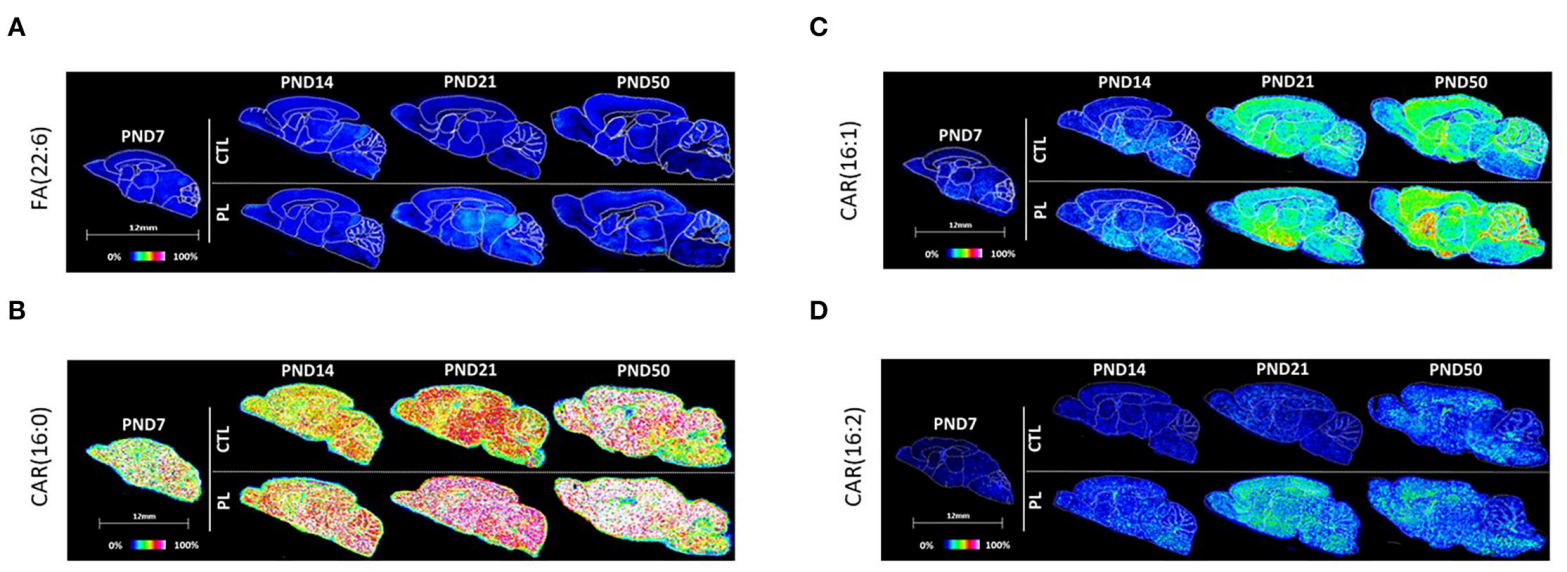
Spatiotemporal distribution of fatty esters. Representative MALDI-MSI images are shown for **(A)** fatty acid FA(22:6) and **(B–D)** acylcarnitines CAR(16:0), CAR(16:1), and CAR(16:2). Control sections are presented on the top. PL-supplemented sections are presented on the bottom. The age is indicated above the images. The signal intensity scale is normalized to 100% of maximum peak intensity across all images.

Fatty acid, 22:6 FA (most probably n-3 docosahexaenoic acid isomer), was the only species annotated for the fatty acyl class. The level of 22:6 FA was rather uniformly distributed across the various brain structures in general. There was an effect of age only at PND 50 with the average signal intensity increased in vST, ST, CTX, HIP, and CBX contrasted by a decrease in the fiber tract-enriched regions, including HB and CC (Figure 5A; Supplementary Figure 3A).

##### Fatty Acyl Carnitine

The second class of fatty acyls identified was the fatty acyl carnitine, which is a fatty acid mitochondrial transporter of FA. Of the 3 species annotated for fatty acyl carnitine, CAR(16:0) and CAR(16:2) showed a similar uniform pattern of distribution across regions and age in general (Figures 5B,D; Supplementary Figures 3B,D). Interestingly, these two species increased significantly with the PL supplementation at all ages and across all structures examined. The expression of the third lipid, CAR(16:1), increased gradually over time in all structures, but the change was less prominent for the fiber tract-enriched regions, HB, AV, FO, and CC (Figure 5C; Supplementary Figure 3C). There was no clear effect of PL for CAR(16:1).

#### Glycerophospholipids

##### Cytidine Diphosphate Diacylglycerol

**Figure 6:**
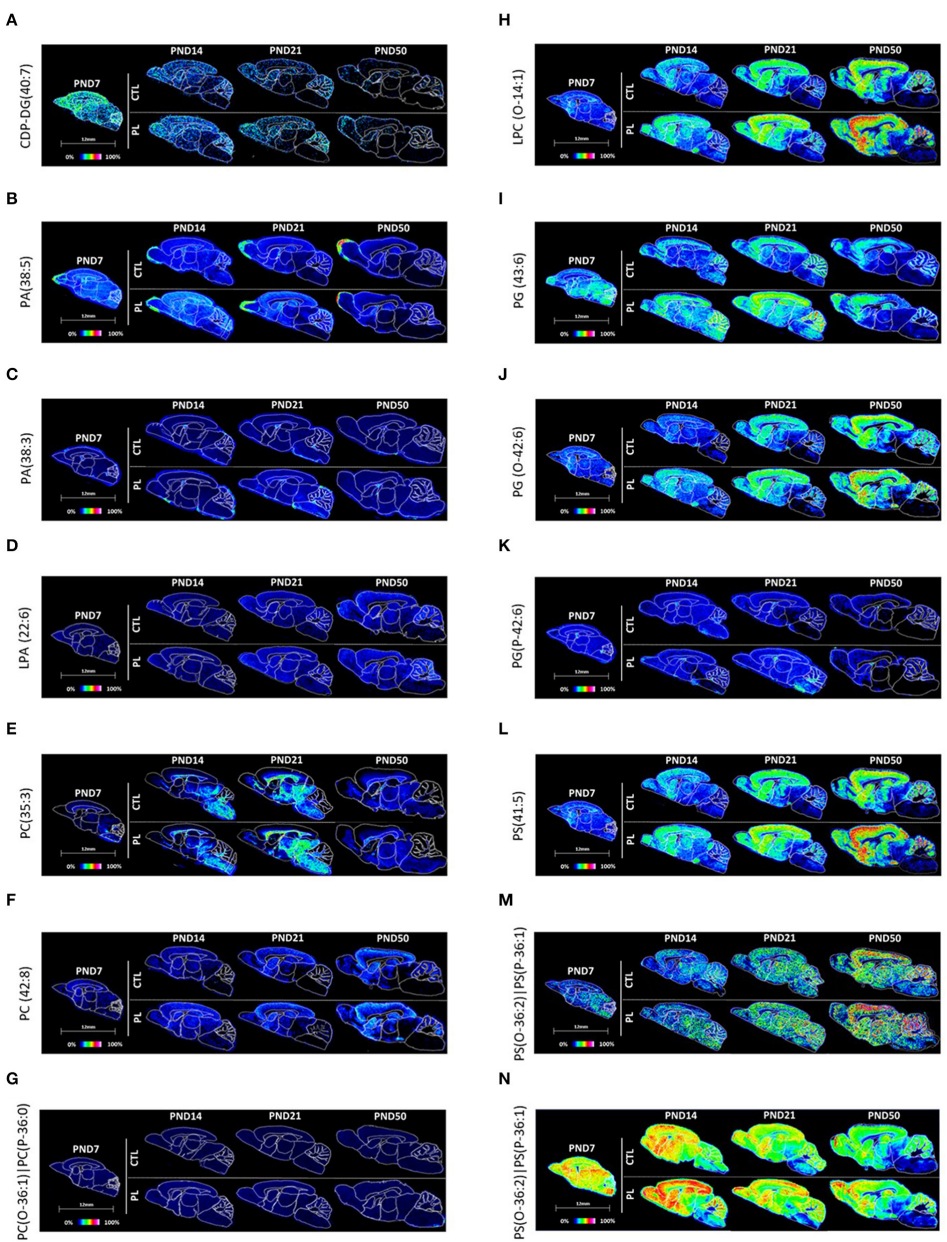
Spatiotemporal distribution of glycerophospholipids. Representative MALDI-MSI images are shown for **(A)** CDP-DG, **(B–D)** glycerophosphates, **(E–H)** glycerophosphocholines, **(I–K)** glycerophosphoglycerols, and **(L–N)** glycerophosphoserines. Control sections are presented on the top. PL-supplemented sections are presented on the bottom. The age is indicated above the images. The signal intensity scale is normalized to 100% of maximum peak intensity across all images.

CDP-DG(40:7) uniformly decreased over time across brain regions with a conserved spatial distribution pattern (Figure 6A; Supplementary Figure 4A). No clear effect of PL was observed for any of the structures.

##### Glycerophosphates

Among the PA, key intermediate of all glycerophospholipids, PA(38:5) was uniformly distributed across all brain regions at PND 7 and showed age-dependent reduction in the signal intensity, which was more pronounced in the caudal part of the brain, such as HYP, TH, MB, and HB (Figure 6B; Supplementary Figure 4B). Conversely, PA(38:3) showed a continuous increase in the signal intensity over time, particularly in OA, ST, vST, CTX, HIP, and CBX (Figure 6C; Supplementary Figure 4C). An opposite pattern was observed for the fiber tract-enriched structures, HB, AV, FO, and CC, where the PA(38:3) level was lower in PND 50 compared to PND 7. Similar to CDP-glycerols, no clear effect of PL was observed for any of the brain regions.

##### Lysophosphatidic Acid

LPA(22:6), which is the monoacylated form of PA, was uniformly expressed across different brain regions in general and showed an age-dependent increase particularly in CBX (Figure 6D; Supplementary Figure 4D). In contrast, the fiber tract-enriched HB, AV, FO, and CC showed a reduction in the level over time. No clear effect of PL was observed for any of the brain regions.

##### Glycerophosphocholine

In total, three types of phosphatidylcholines were annotated: PC(35:3), PC(42:8), and PC(O-36:1)|PC(P-36:0) (Figures 6E–G; Supplementary Figures 4E-G). A high level of PC(35:3) was detected in caudal brain structures, such as HYP, TH, MB, and HB, as well as the fiber tract-enriched AV, FO, and CC. The developmental profile of the PC(35:3) showed an inverted U-shape, where the peak expressions were observed around PND 14 and 21. PC(42:8) showed a complementary distribution to PC(35:3), where a high signal intensity for PC(42:8) was detected particularly in ST, vST, CTX, and HIP, which also showed age-dependent increase. The level in CBX drastically increased at PND 50. In the fiber tract-enriched HB, AV, FO, and CC, there were much lower levels of PC(42:8), which progressively decreased over time. Unlike these phosphatidylcholines, the levels of PC(O-36:1)|PC(P-36:0) did not change across different brain structures or across age groups. Although no clear effect of PL could be observed for PC(35:3) and PC(42:8), PC(O-36:1)|PC(P-36:0) showed a slight increase in rostral regions of the brain, such as OA, ST, vST, CTX, and HIP.

##### Lysophosphocholine

LPC(O-14:1) was similarly distributed throughout the brain at PND 7 and showed an increasing trend over time in all structures (Figure 6H; Supplementary Figure 5A). The levels of LPC(O-14:1) increased with the PL treatment in all brain structures and for all age groups.

##### Glycerophosphoglycerol

The distribution of PG(43:6) and its diacyl counterpart, plasmenyl-glycerol PG(0-42:6), and PG(P-42:6) were relatively homogenous across various brain structures PND 7 (Figures 6I–K; Supplementary Figures 5C–E). The level of PG(43:6) stayed elevated in OA, ST, vST, CTY, and HIP, whereas other structures showed gradual decrease over time, resulting in a heterogenous distribution by PND 50. PG(P-42:6) had a similar reduction in the level, but in all brain structures. In contrast, the level of PG(0-42:6) increased in the rostral structures and CBX, while decreased in the fiber tract-enriched regions, HB and CC, over time, giving rise to a heterogenous distribution by PND 50. Of the three PGs, PG(43:6) showed the most prominent effect by PL in all structures across age groups.

##### Glycerophosphoserines

PS(41:5), PS(O-36:3)|PS(P-36:2), and PS(O-36-3)|PS(P-36-2) were relatively homogeneously distributed at PND 7 (Figures 6L–N; Supplementary Figures 5E-G). The level of PS(41:5) increased in OA, ST, vST, CTX, HIP, and CBX, decreased in HB and AV, and stayed more or less the same in other structures, resulting in a heterogeneous distribution with higher levels in rostral structures compared to the caudal structures by PND 50. In contrast, the level of PS(O-36:3)|PS(P-36:2) and PS(O-36:2)|PS(P-36:1) gradually decreased in all structures, but to a different extent in different brain structures, resulting in higher levels of these lipids in OA, ST, vST, CTX, HIP, and CBX compared to other areas in a lipid distribution similar to PS(41:5) by PND 50. The effect of PL was clearly observable for all three PSs.

##### Glycerophosphoethanolamine

**Figure 7:**
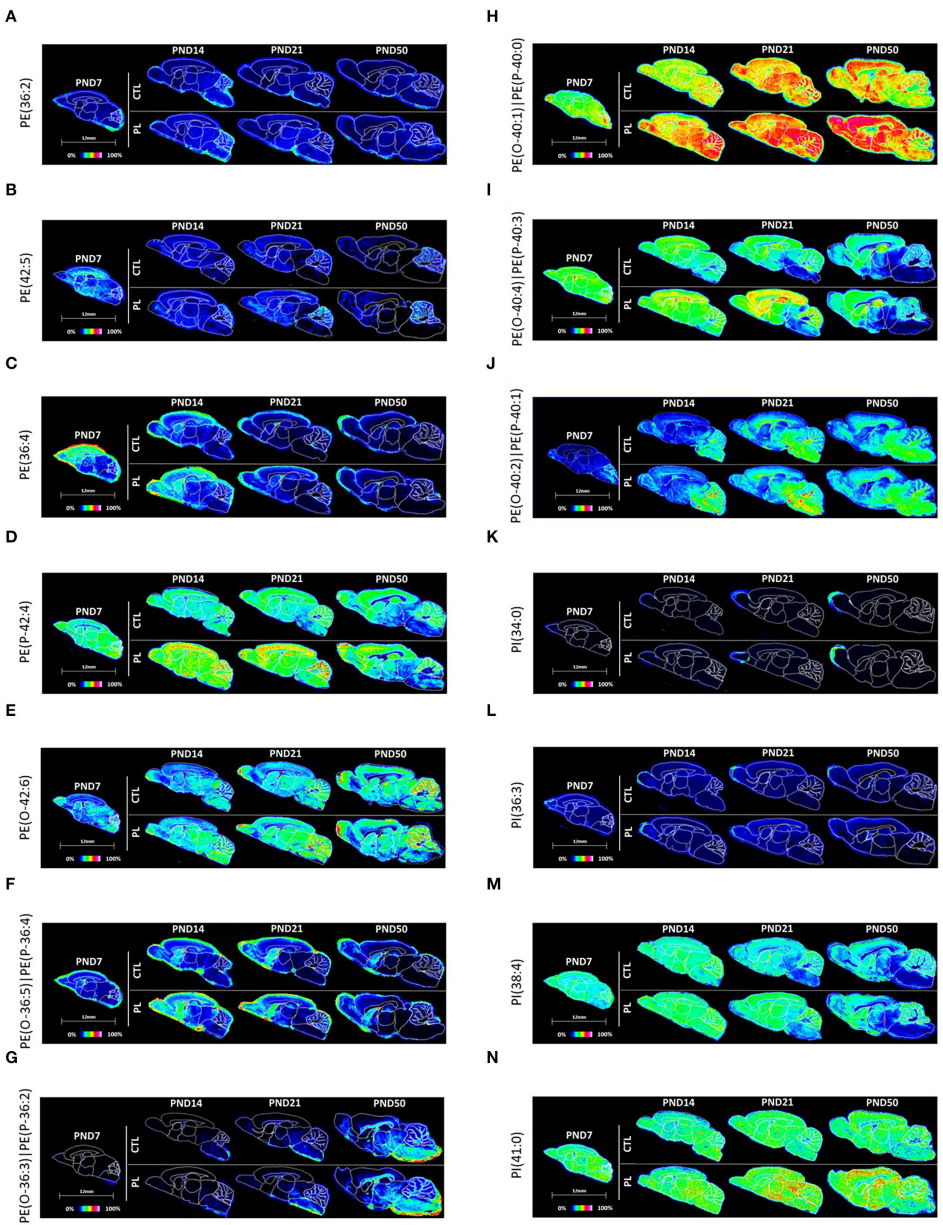
Spatiotemporal distribution of glycerophospholipids. Representative MALDI-MSI images are shown for **(A–J)** phosphoethanolamines and **(K–N)** phosphoinositol. Control sections are presented on the top. PL-supplemented sections are presented on the bottom. The age is indicated above the images. The signal intensity scale is normalized to 100% of maximum peak intensity across all images.

In total, three phosphoethanolamines (PEs) and 7 ether-phosphoethanolamines (ePE, plasmalogen) were annotated. PE(36:2) and PE(42:5) were relatively uniformly distributed across various brain regions, whereas PE(36:4) was preferentially distributed in the rostral areas of the brain, particularly at PND 7 (Figures 7A–C; Supplementary Figures 6A–C). All three PEs exhibited a similar developmental profile, where the average signal intensity reduced over time in almost all brain regions, the pattern of which was most pronounced for PE(36:4) and PE(42:5). The level of PE(42:5) was higher in the CBX compared to other brain regions by PND 14 and 50 due to the lack of developmental decrease in CBX. No clear effect of PL per brain structure was observed for any of these lipids.

All ePEs exhibited a relatively homogeneous distribution across various brain structures at PND 7 (Figures 7D–J; Supplementary Figures 6D–G, 7A–C). However, their developmental profile was rather mixed, which resulted in heterogenous distribution in later time points for majority of ePEs. The decreased PE(P-42:4) signal intensity over time was observed in caudal structures, such as MB and HB, and in fiber tract-enriched structures, AV and FO, but not in the rostral structures, OA, ST, vST, CTX, and HIP. This structure-selective developmental changes in the level of PE(P-42:4) shifted the uniform distribution during early time points compared to more rostral oriented distribution during the later developmental time points. PE(O-36:3)|PE(P-36:2) and PE(O-40:2)|PE(P-40:1) showed a general tendency to increase over time. Interestingly, PE(O-42:6) enrichment seems to be CBX-specific. The developmental increase in the level of PE(O-36:3)|PE(P-36:2) was particularly higher in the caudal structures, such as HB, and fiber tract-enriched AV, FO, and CC compared to the rostral structures, resulting in significant level in the caudal and fiber tracts by PND 50. PE(O-36:5)|PE(P-36:4) and PE(O-40:4)|PE(P-40:3) seemed to decrease over time with a preferential distribution in rostral structures. The level of PE(O-40:1)|PE(P-40:0) did not fluctuate very much over time in any of the structures in general. The effect of PL was most visible for PE(P-42:4), PE(O-40:4)|PE(P-40:3), and PE(O-40:1)|PE(P-40:0) in various structures.

##### Glycerophosphoinositols

All 4 PIs showed a relatively homogenous distribution across all brain structures at PND 7 (Figures 7K–N; Supplementary Figures 7D–G). The signal intensity of PI(34:0), PI(36:3), and PI(38:4) was reduced at a much greater extent in the fiber tract-enriched HB, AV, FO, and CC, resulting in heterogeneous expression by PND 50 accompanied by a lower signal intensity in these structures compared to the rest of the brain. PI(41:0) showed a mixed developmental pattern depending on the brain structure, where the levels increased in CTX and decreased in HB and AV. The level of PI(38.4) was particularly high compared to the other 3 PIs in all structures, where the signal intensity was about more than 100-folds greater than PI(41:0) and more than 10-folds greater than PI(36:3) and PI(34:0). No clear effect of PL was observed for any of the lipids in the different analyzed brain regions.

#### Sphingolipids

##### Phosphosphingolipids

**Figure 8:**
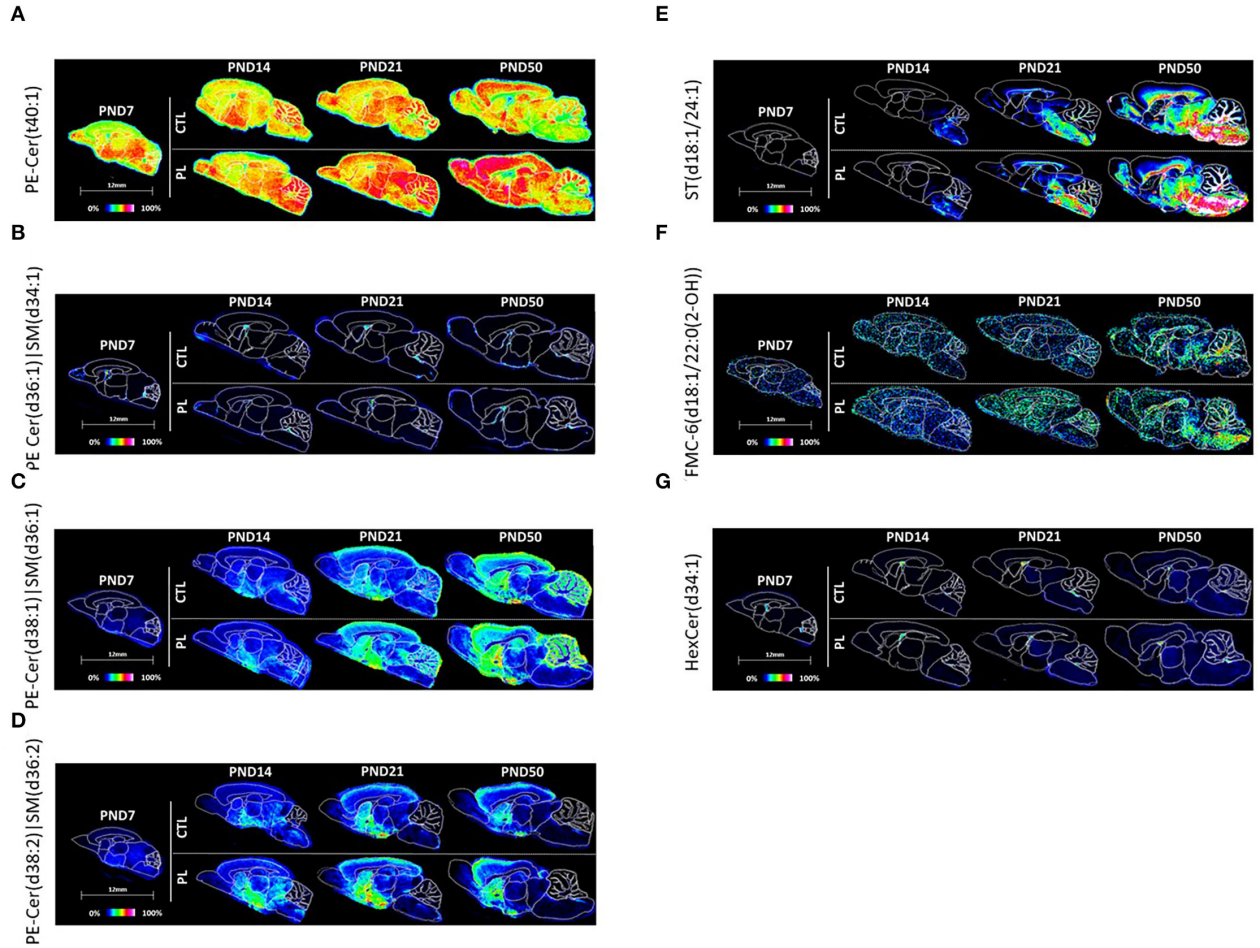
Spatiotemporal distribution of sphingolipids. Representative MALDI-MSI images are shown for **(A–D)** phosphosphingolipids, **(E)** acidic and **(F,G)** neutral glycosphingolipids. Control sections are presented on the top. PL-supplemented sections are presented on the bottom. The age is indicated above the images. The signal intensity scale is normalized to 100% of maximum peak intensity across all images.

PE-Cer(t40:1) and PE-Cer(d36:1)|SM(d34:1) seemed to be homogeneously distributed across all brain structures, and no clear developmental profile was observed except in HB, AV, and FO for PE-Cer(t40:1) and AV for PE-Cer(d36:1)|SM(d34:1) where a slight reduction was observed over time (Figures 8A,B; Supplementary Figures 8A,B). There was a high expression of PE-Cer(d36:1)|SM(d34:1) in the choroid plexus also observed by Martinez et al. (13). The level of PE-Cer(d38:1)|SM(d36:1) was homogeneously distributed in the brain at PND 7 and gradually increased in all structures to different degrees, resulting in a higher level in ST, vST, CTX, HIP, HYP, and CBX at PND 50 compared to other structures (Figure 8C; Supplementary Figure 8C). In contrast, the level of PE-Cer(d38:2)|SM(d36:2) was higher in HYP, TH, MB, and HB at PND 7. Interestingly, a unique distribution pattern for Cer(d38:2)|SM(d36:2) emerged compared to other category and classes of lipids, where higher levels were detected in ST, vST, and HYP compared to other structures, especially at PND 14 and 21 (Figure 8D; Supplementary Figure 8D). Among all PEs, PE-Cer(t40:1) showed a very clear effect by PL in all structures across all ages.

##### Acidic and Neutral Glycosphingolipids

The acidic glycosphingolipid, sulfatide ST(d18:1/24:1), was homogeneously expressed at PND 7 at a very low level, but increased exponentially at PND 50 in all structures, especially for the mid to caudal parts of the brain, HYP, TH, MB, and HB, and the fiber tracts, AV, FO, and CC at PND 50 (Figure 8E; Supplementary Figure 8E). This heterogenous changes in the level in various structures over time resulted in a caudal brain structure and fiber tract heavy distribution at PND 21 and 50.

The distribution of the neutral glycosphingolipids, FMC-6(d18:1/22:0(2-OH)), and HexCer(d34:1) was relatively homogenously distributed across all brain structures at PND 7 (Figures 8F,G; Supplementary Figures 8F,G). For FMC-6(d18:1/22:0(2-OH)), the signal intensity was selectively increased in HYP, TH, MB, HB, AV, FO, and CC at PND 50, resulting in a caudally and fiber tract-focused distribution at PND 50. The signal intensity of the galactocerebroside, HexCer(d34:1), was too variable to clearly observe a developmental pattern. No clear effect of PL supplementation was observed per structure for any of these glycosphingolipids.

### Summary of the Developmental Profile of 39 Lipids for Each ROI

**Figure 9:**
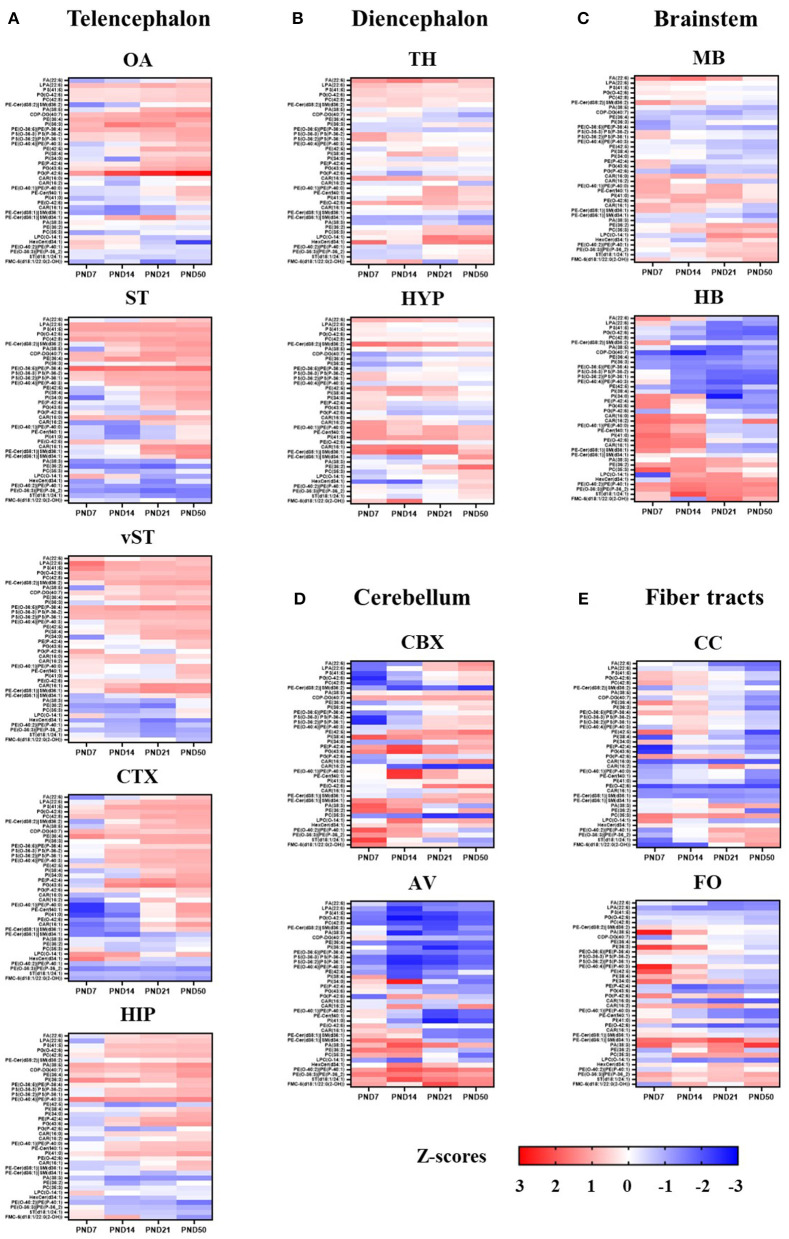
Summary of developmental profile of 39 lipids in each ROI. Developmental profile of 39 lipids in each ROI is represented as a heat map. The scale indicates lowest z-score (-3) in dark blue to highest z-score (3) in dark red. The lipids with coordinated spatio-temporal distribution were clustered together based on the unsupervised pattern recognition analysis using HCA. ROIs are grouped according to the following brain divisions: Telencephalon **(A)**, Diencephalon **(B)**, Brain stem **(C)**, Cerebellum **(D)** and Fiber Tracts **(E)**. OA, olfactory area; ST, striatum; vST, ventral striatum; CTX, cortex; HIP, hippocampus; TH, thalamus; HYP, hypothalamus; MB, midbrain; HB, hindbrain; CBX, cerebellum; AV, arbor vitae; CC, corpus callosum; FO, fornix.

The lipids with coordinated spatiotemporal distribution were clustered together based on unsupervised pattern recognition using HCA to create the summary of heatmaps for all 38 lipids for each ROIs (Figure 9). A clear developmental pattern depending along a rostral–caudal axis of the structures and fiber tract content emerged as result of this analysis. The telencephalic rostral structures, OA, ST, vST, CTX, and HIP, showed a similar set of lipid species that had coordinated spatiotemporal distribution across different time points (Figure 9A). Similarly, the spatiotemporal distributions of lipids in diencephalic structures, TH and HYP, were more similar to each other than the telencephalic structures, CBX, or fiber tract-enriched structures (Figure 9B). The brainstem structures and fiber tract structures seemed to also share a set of lipids that are spatiotemporally associated (Figures 9C–E). The only exception was CBX, where the spatiotemporal distribution of the lipids did not follow similar pattern as observed for other brain areas (Figure 9D).

## Discussion

Nutrition plays a critical role in promoting brain development during early life (24). In this study, we examined (1) the general developmental changes in brain lipidome and (2) the effect of PL supplementation, one of the major components of the MFGM in human breast milk, on brain lipid composition during development.

Regarding the developmental changes in brain lipidome, we observed that almost a half of annotated lipids gradually increased and peaked at PND 50, whereas the other lipids either decreased or stayed approximately the same over time. More specifically, within the time-period investigated, the age-dependent changes in the lipid distribution were most prominent at PND 21 and 50. For example, fatty acyl CAR(16:1) and sphingolipid PE-Cer(d38:1)|SM(d36:1) gradually increased over time in all structures, whereas glycerophospholipids, such as CDP-DG(40:7) and PE(36:4), gradually decreased over time. Other glycerophospholipids, such as ether phosphatidylcholine PC(O:36:1)|PC(P-36:0) and ether phosphatidylethanolamine PE(O-40:1)|PE(P-40:0), stayed more or less constant across the age groups studied. These results show that the brain lipidome is changing heterogeneously along time during brain development.

The selected time points (PND 7, 14, 21, 50) in this study correspond to key developmental events during rat brain development conserved among different mammalian species, including gliogenesis, axonal and dendritic growth and synapse formation (61). At PND 21, the rat brain reaches 95% of its adult weight, which is accompanied by the higher synaptic density and myelination rate, comparable to a human brain development at 2–3 years of age (61). Similarly, from PND 21 to 50, the volume of gray matter and cortical thickness reaches its maximum; cortex neural networks structurally mature; and synaptic density begins to decrease, which parallels the increase in the white matter, reflecting ongoing myelination (61). Accordingly, PND 7 to 21 in rodents corresponds to infancy and up to PND 50 to adolescence in human in terms of brain maturation, in agreement with the developmental hallmarks of brains as previously described (61, 62). Therefore, the prominent divergence of brain lipidome observed in our study at PND 21 and PND 50 may reflect the specialization of neural networks that occurs during the final stages of brain maturation. Nutritional interventions enriched in PL, during this time period, might be beneficial during brain development and support brain function later in life (38).

Among the molecular ion peaks whose levels were significantly increased by PL supplementation in the whole brain, we were able to annotate 39 lipids, which belonged to fatty acyl, glycerophospholipid, and sphingolipid categories. The key intermediate molecule in glycerophospholipids biosynthesis species, phosphatidic acid [PA(38:5) and PA(38:3)], and its conversion product, cytidine diphosphate-diacylglycerol (CDP-DG) such as CDP-DG(40:7)), were also increased in the total brain following PL supplementation. Moreover, phosphatidic acid is a key precursor for all phospholipid synthesis, including PC, PE, and PS (63), whereas CDP-DG is involved in the pathway of biosynthesis of acidic phospholipids (64). CDP-DG can react with inositol to form phosphatidylinositol (PI) or with sn-glycerol-3-phosphate to form phosphatidyl glycerophosphate, which can then be converted to cardiolipin and subsequently forms a complex with PG (43:6). Although the actual functions of PA(38:5) and PA(38:3) in brain development have not been characterized, other PA species, such as PA(38:1) and PA(40:6), have been reported to be synthetized during neurosecretion and neurite outgrowth (65). Similarly, lysophospholipids also serve as precursors for *de novo* biosynthesis of more complex glycerophospholipids. For example, because of it acyl chain composition, LPA (22:6) could influence the content of long-chain polyunsaturated fatty acids (LC-PUFAs) in cell membrane (66). Interestingly, non-esterified DHA, FA (22:6), previously reported as the major source of brain DHA, seems to share similar developmental pattern in, gray matter of the cerebellum where its level increased with age. Such accumulation might be related to the generation of new synapses in cerebellar cortex in response to fine motor skill learning (67–69).

Similarly, when the levels of lipids were analyzed for specific brain regions, the effect of PL was prominent for a specific set of lipids comprising fatty esters [e.g., CAR(16:0) and CAR(16:2)], glycerophosphocholines [e.g., PC(O-36:1)|PC(P-36:0) and LPC(O-14:1)], ether-phosphoethanolamines (plasmalogen) [e.g., PE(O-40:1)|PE(P-40:0) and PE(O-40:2)|PE(P-40:3)] and phosphosphingolipids [e.g., PE-Cer(t40:1)]. These molecules show relatively steady levels during development, suggesting that their levels accompany brain growth and contribute to the overall brain growth as critical structural components of brain development. Interestingly, few other lipids showed age-dependent increase or decrease that do not follow the overall dynamic and contribute to specific biological processes, such as myelination. For example, PE(O-36:3)|PE(P-36:2) and ST(d18:1/24:1) overall increased from PND 7 to PND 50 in all brain structures, but more intensively in the caudal brain areas, such as HYP, TH, MB, and HB, and in fiber tract-enriched AV, FO, and CC. Plasmalogen has been shown to increase dramatically with the level of myelination during brain development in humans (70). Similarly, the level of sulfatides was reported to increase during a period of high demand for myelination during brain development (71) and ST(d18:1/24:1) was specifically mapped in white matter regions (13).

Interestingly, species belonging to sphingomyelin show diverse spatiotemporal patterns. This diversity seems to stem from the fact that there are two functionally distinct types of sphingomyelins, one involved in general brain development (e.g., PE-Cer(t40:1) and the other for telencephalic development (e.g., PE-Cer(d38:1)|SM(d36:1), PE-Cer(d38:2)|SM(d36:2), particularly in the gray matter. As such, PE-Cer(t40:1) showed a ubiquitous spatial distribution throughout the development, which might imply a continuous requirement of such species in the cell membranes of neural cells. In contrast, PE-Cer(d38:1)|SM(d36:1) and PE-Cer(d38:2)|SM(d36:2) were particularly enriched in the telencephalon and gray matter. Sphingomyelin (phosphatidylcholine-ceramide) or its analog, phosphatidylethanolamine-ceramide, are synthetized by translocation of phosphocholine or ethanolamine from PE or PC to ceramides in brain and especially in the synaptic membrane (72). Both types of molecules are involved in neuronal differentiation and synaptic transmission in neuronal-glial connection and are also associated with myelin stability (3). Thus, enrichment of sphingomyelin in telencephalon and cerebellum suggests a role in supporting these neurodevelopmental processes in these regions.

In addition to sphingomyelin, we have annotated other lipid species with high number of unsaturation that are preferentially distributed in telencephalic regions, such as PA(38:5), PA(38:3), LPA(22:6), PG(O-42:6), PC(42:8) and PS (41:5). In fact, we noted that brain structures with similar functions or common developmental origins showed coordinated lipid specie distributions. For example, CTX, HIP, and ST, which form the telencephalon, showed a coordinated spatiotemporal lipid distribution compared to, for example, diencephalic structures in our study. The telencephalic structures tend to develop later in comparison with, for example, the brainstem, which is the most mature structure at birth because of its critical role in regulating essential vital functions common to mammalian species (73). Therefore, the age-dependent profile of lipid species in various brain regions may reflect the different timing of critical biological processes needed during the development of respective brain structures. It also suggests that the reason behind the heterogeneous effect of the PL supplement may be, in part, due to the different timing of the lipid requirements to support the differential maturation of the various brain structures during the development.

It is noteworthy that we observe that acylcarnitines (CARs) levels are elevated in the PL-supplemented animals. CARs are energetic molecules which serve as carriers to transport activated long-chain fatty acids into mitochondria that are metabolized through β-oxidation to serve as a major source of energy for cell activities. Palmitoyl-L-carnitine (CAR 16:0), more specifically, has been shown to be involved in phospholipid and fatty acid turnover, influencing SM, PS, and PC composition, respectively, through palmitate incorporation (74). Finally, we have also observed that some lipid species are not necessarily elevated or even reduced. Taken together, our findings suggest that the lipid supplement acting as a source of energy for cells alone is not sufficient to explain the observed lipidomic pattern in this study. Instead, it is likely a result of a complex orchestration of lipid homeostasis regulating various structural, functional, and cellular energy processes needed during early brain development.

Finally, we also annotated lipid species that have not been previously reported to our knowledge, including wax monoesters, phosphoinositolglycans, and mannosyl-inositol-phosphoryl-ceramides (data not shown). Wax monoesters have been previously identified in sebum of the skin (75). One group has reported that wax ester was detected using thin-layer chromatography in homogenates of rat brain (76), but since then, this is the first study confirming its presence. Wax monoester and ether-lipids are downstream products of fatty alcohol, which is a reduced product from fatty acid by fatty-acyl-CoA reductases (77). Therefore, it is likely that wax synthases exist in the brain. Phosphoinositolglycans and ceramide phosphoinositols have been reported in yeast and bacteria (78, 79). It is unlikely that these findings are the results of an artifact of experimental procedure because wax monoesters levels, in particular, seemed to increase with the PL supplementation. Instead, the absence of wax monoesters detections in previous reports could be explained by the narrow m/z ranges selected compared to our study. While previous studies use m/z ranges between 500 and 1,200, we specifically decide to use the m/z range between 50 and 2,000 to enhance our chance to detect additional lipid species (13). Nevertheless, their specific roles in the brain will require further investigation, which is out of scope of the current study.

## Conclusions

In conclusion, we described for the first time a dynamic lipidome signature during rodent brain development with region-specific hallmarks. We also demonstrated that this signature could be influenced by a chronic PL supplementation. Indeed, additional PL dietary intake modulates the brain lipid composition during early development. Finally, we further provided evidence that various lipid species have specific temporal and spatial patterns, which may reflect underlying age-related and structure-specific neurodevelopmental processes. Together, these results suggest that dietary PL supplementation could support early brain development.

## Data Availability Statement

The raw data supporting the conclusions of this article will be made available by the authors, without undue reservation.

## Ethics Statement

The animal study was reviewed and approved by Direction Régionale de l'Alimentation, de l'Agriculture et de la Forêt du Languedoc- Roussillon - France-(agreement #A 34-169-002; May 02, 2014).

## Author Contributions

MO designed, analyzed and interpreted the experiments, and drafted the manuscript. KK supported the interpretation of the data and writing of the manuscript. AR ran the statistical analysis. FG, SS, PS, and NS provided input during study execution and result interpretation and revised the manuscript. AP developed the methodology to extract the polar lipids. MG directed the study for ImaBiotech and performed lipid annotation. AT analyzed the images and prepared the figures. All authors contributed to the preparation of the manuscript and agreed on the final version.

## Funding

This study received funding from Société des Produits Nestlé S.A. The funder had the following involvement with the study: Study design, data analysis, decision to publish, and preparation of the manuscript.

## Conflict of Interest

MO, KK, AR, FG, AP, SS, PS, and NS were employed by the company Société des Produits Nestlé SA. MG and AT were employed by ImaBiotech SAS.

## Publisher's Note

All claims expressed in this article are solely those of the authors and do not necessarily represent those of their affiliated organizations, or those of the publisher, the editors and the reviewers. Any product that may be evaluated in this article, or claim that may be made by its manufacturer, is not guaranteed or endorsed by the publisher.
