# Supplementary material for: Early Life to Adult Brain Lipidome Dynamic: A Temporospatial Study Investigating Dietary Polar Lipid Supplementation Efficacy
Source: Front Nutr. 2022 Jul 26;9:898655. doi: 10.3389/fnut.2022.898655 (PMC9364220; doi:10.3389/fnut.2022.898655)
Supplement: Supplementary file 1 [file Table_1.DOCX]

| **Ingredient (g/kg)** | **AIN-93G** | **AIN-93M** |
| --- | --- | --- |
| Casein | 200 | 140 |
| Fat | 70 | 40 |
| Cellulose | 50 | 50 |
| Cornstarch | 397.5 | 466 |
| Maltodextrin | 132 | 155 |
| Sucrose | 100 | 100 |
| AIN-93 Mineral mix | 35 | 35 |
| AIN-93 Vitamin mix | 10 | 10 |
| L-Cystine | 3 | 1.8 |
| Choline bitartrate | 2.5 | 2.5 |
| t-Butylhydroquinone | 0.014 | 0.008 |
| Proteins (% energy) | 19.3 | 14.1 |
| Carbohydrate (% energy) | 64 | 75.9 |
| Lipids (% energy) | 16.7 | 10 |
| Energy content (Kcal/kg) | 3.76 | 3.60 |

**Supplementary Table 1**. **Composition and energy content of basal AIN-93 growth (G) and maintenance (M) purified rodent diets.**
