# Supplementary material for: Early Life to Adult Brain Lipidome Dynamic: A Temporospatial Study Investigating Dietary Polar Lipid Supplementation Efficacy
Source: Front Nutr. 2022 Jul 26;9:898655. doi: 10.3389/fnut.2022.898655 (PMC9364220; doi:10.3389/fnut.2022.898655)
Supplement: Supplementary file 2 [file Table_2.docx]

| **Molecular species** | **Content** |  | **Molecular species** | **Content** |  | **Molecular species** | **Content** |  | **Molecular species** | **Content** |
| --- | --- | --- | --- | --- | --- | --- | --- | --- | --- | --- |
| **SM(30:1)** | 0.3 |  | **PC(28:0)** | 1.4 |  | **PE(32:0)** | 0.7 |  | **PI(32:0)** | 1.2 |
| **SM(32:0)** | 1.3 |  | **PC(30:0)** | 6.7 |  | **PE(32:1)** | 1.6 |  | **PI(32:1)** | 1.0 |
| **SM(32:1)** | 6.8 |  | **PC(30:1)** | 0.5 |  | **PE(32:2)** | 0.5 |  | **PI(34:0)** | 1.7 |
| **SM(34:0)** | 3.3 |  | **PC(32:0)** | 10.5 |  | **PE(34:0)** | 1.1 |  | **PI(34:1)** | 7.9 |
| **SM(34:1)** | 23.6 |  | **PC(32:2)** | 0.9 |  | **PE(34:1)** | 8.8 |  | **PI(34:2)** | 1.0 |
| **SM(34:2)** | 0.9 |  | **PC(32:6)** | 0.2 |  | **PE(34:2)** | 6.4 |  | **PI(34:3)** | 0.3 |
| **SM(35:0)** | 0.5 |  | **PC(34:0)** | 6.3 |  | **PE(35:0)** | 0.2 |  | **PI(35:1)** | 1.4 |
| **SM(35:1)** | 2.1 |  | **PC(34:1)** | 16.3 |  | **PE(35:1)** | 1.2 |  | **PI(35:2)** | 0.6 |
| **SM(35:2)** | 0.2 |  | **PC(34:2)** | 7.3 |  | **PE(35:2)** | 1.2 |  | **PI(36:0)** | 3.5 |
| **SM(36:0)** | 0.6 |  | **PC(34:3)** | 1.6 |  | **PE(35:3)** | 0.4 |  | **PI(36:1)** | 30.2 |
| **SM(36:1)** | 3.6 |  | **PC(35:0)** | 0.9 |  | **PE(36:0)** | 1.6 |  | **PI(36:2)** | 26.3 |
| **SM(36:2)** | 0.5 |  | **PC(35:1)** | 2.1 |  | **PE(36:2)** | 40.5 |  | **PI(36:3)** | 7.3 |
| **SM(37:0)** | 0.3 |  | **PC(35:2)** | 1.2 |  | **PE(36:3)** | 17.3 |  | **PI(36:4)** | 1.3 |
| **SM(37:1)** | 1.0 |  | **PC(35:3)** | 0.3 |  | **PE(36:4)** | 5.4 |  | **PI(37:2)** | 0.8 |
| **SM(38:0)** | 2.0 |  | **PC(36:0)** | 1.7 |  | **PE(36:5)** | 0.9 |  | **PI(37:3)** | 0.3 |
| **SM(38:1)** | 7.8 |  | **PC(36:1)** | 10.9 |  | **PE(37:0)** | 0.2 |  | **PI(38:1)** | 0.7 |
| **SM(38:2)** | 0.2 |  | **PC(36:2)** | 15.3 |  | **PE(37:1)** | 0.4 |  | **PI(38:2)** | 1.5 |
| **SM(38:7)** | 0.2 |  | **PC(36:3)** | 7.1 |  | **PE(37:2)** | 0.8 |  | **PI(38:3)** | 3.7 |
| **SM(38:8)** | 0.9 |  | **PC(36:4)** | 2.9 |  | **PE(37:3)** | 0.3 |  | **PI(38:4)** | 4.3 |
| **SM(39:1)** | 7.9 |  | **PC(36:5)** | 0.5 |  | **PE(38:1)** | 0.4 |  | **PI(38:5)** | 3.3 |
| **SM(40:0)** | 2.5 |  | **PC(37:2)** | 0.4 |  | **PE(38:2)** | 0.6 |  | **PI(38:6)** | 0.8 |
| **SM(40:1)** | 10.8 |  | **PC(37:3)** | 0.2 |  | **PE(38:3)** | 1.1 |  | **PI(40:4)** | 0.2 |
| **SM(40:2)** | 2.2 |  | **PC(38:0)** | 0.4 |  | **PE(38:4)** | 2.0 |  | **PI(40:5)** | 0.5 |
| **SM(40:6)** | 0.2 |  | **PC(38:1)** | 0.5 |  | **PE(38:5)** | 2.1 |  | **PI(40:6)** | 0.3 |
| **SM(40:7)** | 2.1 |  | **PC(38:2)** | 0.4 |  | **PE(38:6)** | 0.8 |  |  |  |
| **SM(40:9)** | 1.5 |  | **PC(38:3)** | 0.4 |  | **PE(38:8)** | 0.3 |  |  |  |
| **SM(41:1)** | 6.4 |  | **PC(38:4)** | 0.6 |  | **PE(40:4)** | 0.2 |  |  |  |
| **SM(42:0)** | 0.7 |  | **PC(38:5)** | 0.9 |  | **PE(40:5)** | 0.9 |  |  |  |
| **SM(42:1)** | 2.7 |  | **PC(38:6)** | 0.3 |  | **PE(40:6)** | 1.0 |  |  |  |
| **SM(42:2)** | 1.2 |  | **PC(38:8)** | 0.4 |  | **PE(40:7)** | 0.3 |  |  |  |
| **SM(42:7)** | 1.3 |  | **PC(40:6)** | 0.3 |  |  |  |  |  |  |
| **SM(42:9)** | 2.3 |  | **PC(40:7)** | 0.2 |  |  |  |  |  |  |
| **SM(44:7)** | 0.4 |  |  |  |  |  |  |  |  |  |
|  |  |  |  |  |  |  |  |  |  |  |

**Supplementary table 2: Molecular species composition of phospholipids contained in the polar lipid extract.** Total number of carbons and number of unsaturation in the acyl-chains are indicated between parentheses.The lipid components in the table are arranged based on the molecular species composition. Content is expressed as %. SM: Sphyngomyelin, PC: Phosphatidylcholine, PE: Phosphatidylethanolamine, PI: Phosphatidylinositol.
