# Supplementary material for: Early Life to Adult Brain Lipidome Dynamic: A Temporospatial Study Investigating Dietary Polar Lipid Supplementation Efficacy
Source: Front Nutr. 2022 Jul 26;9:898655. doi: 10.3389/fnut.2022.898655 (PMC9364220; doi:10.3389/fnut.2022.898655)
Supplement: Supplementary file 3 [file Table_3.docx]

| **Category** | **Main class** | **Subclass** | **Proposed compound** | **Formula** | **Ion** | **Experimental m/z** | **Theorotical m/z** |
| --- | --- | --- | --- | --- | --- | --- | --- |
| **Fatty Acyls** | Fatty Acids | Fatty acids | FA(22:6) | C22H32O2 | [M-H]- | 327.2275 | 327.233 |
|  | Fatty Esters | Acyl carnitines | CAR(16:0) | C23H45NO4 | [M+H]+ | 400.34023 | 400.3421 |
|  |  |  | CAR(16:2) | C25H45NO4 | [M+H]+ | 424.33844 | 424.3421 |
|  |  |  | CAR(16:1) | C23H43NO4 | [M+Cl]- | 432.2916 | 432.2886 |
| **Glycerophospholipids** | CDP-Glycerols | CDP-diacylglycerol | CDP-DG(40:7) | C52H83N3O15P2 | [M-H]- | 1050.5255 | 1050.5227 |
|  | Glycerophosphates | Diacylglycerophosphate | PA(38:5) | C41H71O8P | [M-H]- | 721.4836 | 721.4814 |
|  |  |  | PA(38:3) | C41H75O8P | [M-H]- | 725.5105 | 725.5127 |
|  |  | Monoacylglycerophosphate | LPA(22:6) | C25H39O7P | [M-H]- | 481.2398 | 481.2361 |
|  | Glycerophosphocholine | Ether-Phosphocholine | PC(O-36:1)\|PC(P-36:0) | C44H88NO7P | [M+H]+ | 774.634 | 774.6371 |
|  |  | Phosphocholine | PC(35:3) | C43H80NO8P | [M+Cl]- | 804.5318 | 804.5316 |
|  |  |  | PC(42:8) | C50H84NO8PK | [M+K]+ | 896.54784 | 896.5566 |
|  |  | Ether-Lysophosphocholine | LPC(O-14:1) | C22H46NO6P | [M+H-H2O]+ | 434.30435 | 434.303 |
|  | Glycerophosphoglycerol | Ether-Phosphoglycerol | PG(P-42:6) | C48H83O9P | [M-H]- | 833.5781 | 833.5702 |
|  |  |  | PG(O-42:6) | C48H85O9PK | [M+K]+ | 875.55472 | 875.5563 |
|  |  | Phosphoglycerol | PG(43:6) | C49H85O10P | [M+H-H2O]+ | 847.58517 | 847.5847 |
|  | Glycerophosphoserines | Ether-Phosphoserine | PS(O-36-3)\|PS(P-36-2) | C42H78NO9P | [M+H]+ | 772.55441 | 772.5487 |
|  |  |  | PS(O-36:2)\|PS(P-36:1) | C42H80NO9P | [M+H]+ | 774.5598 | 774.5643 |
|  |  | Phosphoserine | PS(41:5) | C47H82NO10PNa | [M+Na]+ | 874.55438 | 874.5569 |
|  | Glycerophosphoethanolamine | Phosphoethanolamine | PE(36:2) | C41H78NO8P | [M-H]- | 742.5447 | 742.5392 |
|  |  |  | PE(36:4) | C41H74NO8P | [M-H]- | 738.5123 | 738.5079 |
|  |  |  | PE(42:5) | C47H84NO8P | [M-H]- | 820.5928 | 820.5862 |
|  |  | Ether-Phosphoethanolamine | PE(P-42:4) | C47H86NO7PK | [M+K]+ | 846.57958 | 846.5773 |
|  |  |  | PE(O-42:6) | C47H84NO7PK | [M+K]+ | 844.56832 | 844.5617 |
|  |  |  | PE(O-36:5)\|PE(P-36:4) | C41H74NO7P | [M-H]- | 722.5193 | 722.513 |
|  |  |  | PE(O-36:3)\|PE(P-36_2) | C41H78NO7P | [M-H]- | 726.5451 | 726.5443 |
|  |  |  | PE(O-40:1)\|PE(P-40:0) | C43H86NO7PK | [M+K]+ | 798.57918 | 798.5773 |
|  |  |  | PE(O-40:4)\|PE(P-40:3) | C45H84NO7PK | [M+K]+ | 820.5687 | 820.5617 |
|  |  |  | PE(O-40:2)\|PE(P-40:1) | C45H88NO7PK | [M+K]+ | 824.59761 | 824.593 |
|  | Glycerophosphoinositols | Phosphoinositol | PI(34:0) | C43H83O13P | [M-H]- | 837.5516 | 837.5499 |
|  |  |  | PI(36:3) | C45H81O13P | [M-H]- | 859.5312 | 859.5342 |
|  |  |  | PI(38:4) | C47H82O13P | [M-H]- | 885.5499 | 885.5481 |
|  |  |  | PI(41:0) | C50H97O13PK | [M+K]+ | 975.63143 | 975.6298 |
| **Sphingolipids** | Phosphosphingolipids | Phosphoethanolamine-Ceramide | PE-Cer(t40:1) | C42H85N2O7PK | [M+K]+ | 799.57248 | 799.5726 |
|  |  | Phosphoethanolamine-Ceramide\| Sphingomyelin | PE-Cer(d36:1)\|SM(d34:1) | C38H77N2O6P | [M-H]- \|[M-CH3]- | 687.5433 | 687.5447 |
|  |  |  | PE-Cer(d38:2)\|SM(d36:2) | C40H79N2O6P | [M-H]- \| [M-CH3]- | 713.565 | 713.5603 |
|  |  |  | PE-Cer(d38:1)\|SM(d36:1) | C40H81N2O6P | [M-H]- \| [M-CH3]- | 715.5793 | 715.576 |
|  | Acidic glycosphingolipids | Sulfatide | ST(d18:1/24:1) | C48H91NO11S | [M-H]- | 888.624 | 885.5481 |
|  | Neutral glycosphingolipids | Hexosyl-ceramide | HexCer(d34:1) | C40H77NO8 | [M-H]- | 698.5514 | 698.5576 |
|  |  | Fast-migrating ceramide | FMC-6(d18:1/22:0(2-OH)) | C56H99NO14 | [M+Cl]- | 1044.682 | 1044.675958 |

**Supplementary Table 3. Annotated Lipid species.**
