# Supplementary figures and images for: Early Life to Adult Brain Lipidome Dynamic: A Temporospatial Study Investigating Dietary Polar Lipid Supplementation Efficacy
Source: Front Nutr. 2022 Jul 26;9:898655. doi: 10.3389/fnut.2022.898655 (PMC9364220; doi:10.3389/fnut.2022.898655)

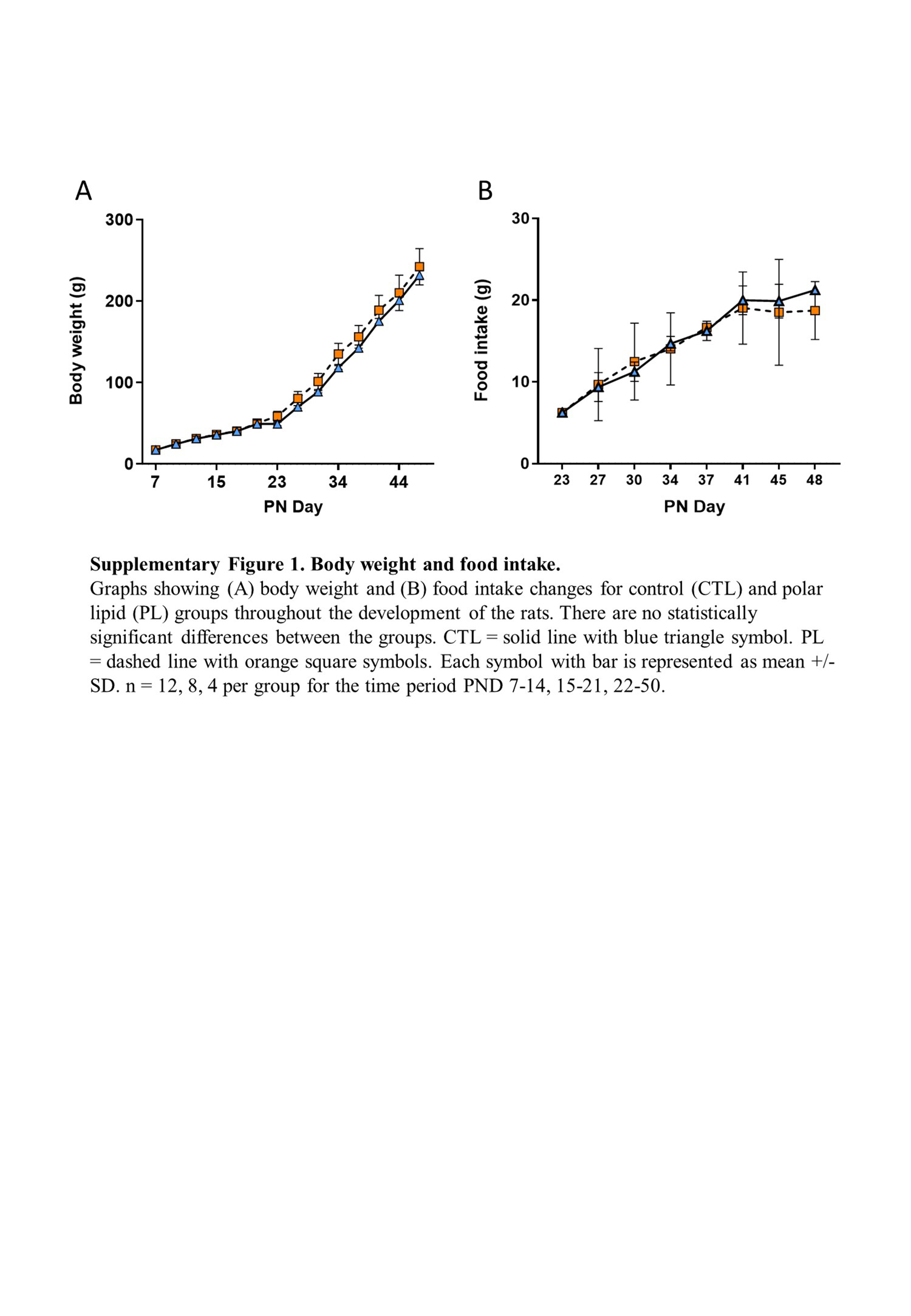

Supplement: Supplementary file 4 [file Image_1.JPEG]

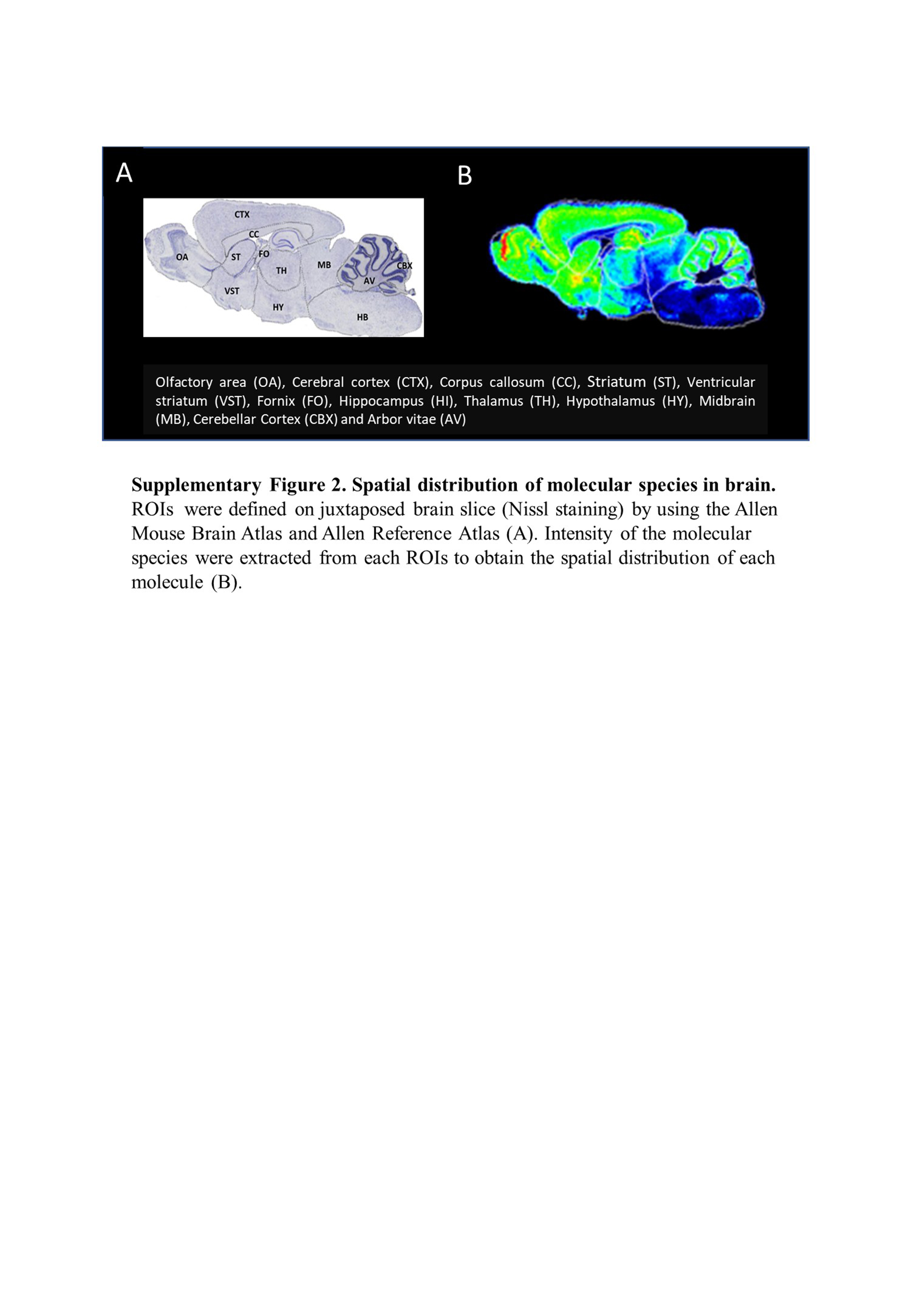

Supplement: Supplementary file 5 [file Image_2.JPEG]

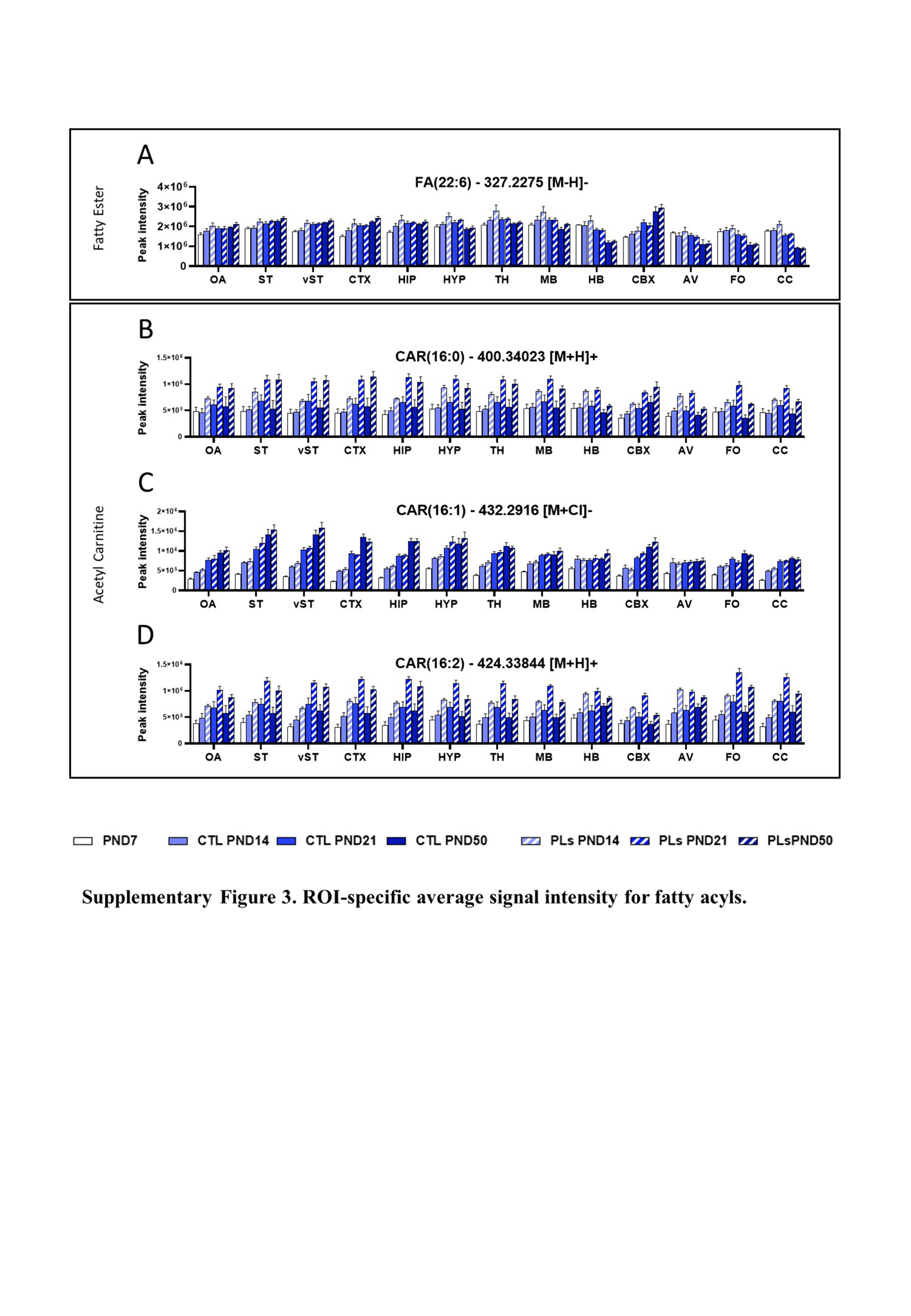

Supplement: Supplementary file 6 [file Image_3.JPEG]

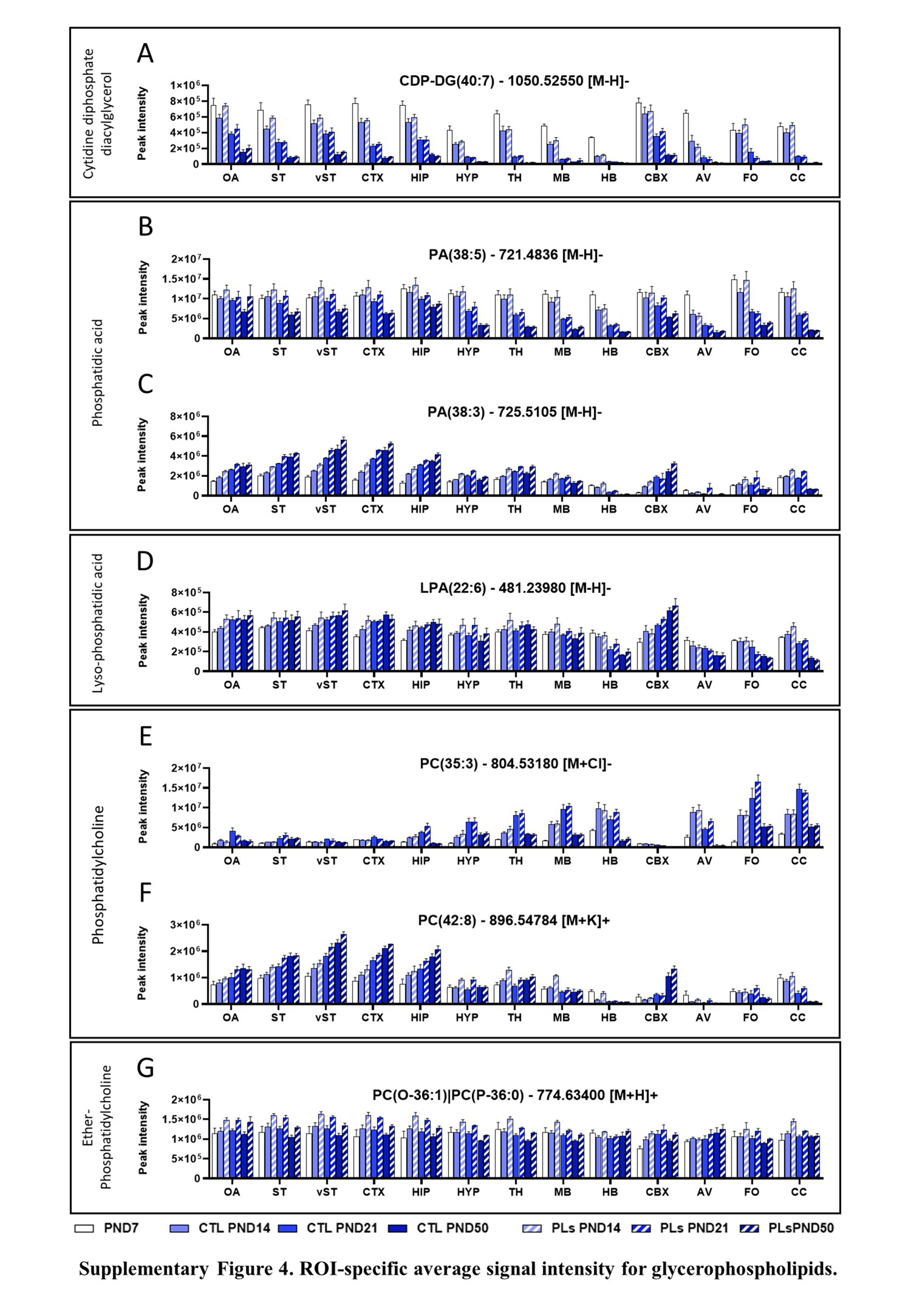

Supplement: Supplementary file 7 [file Image_4.JPEG]

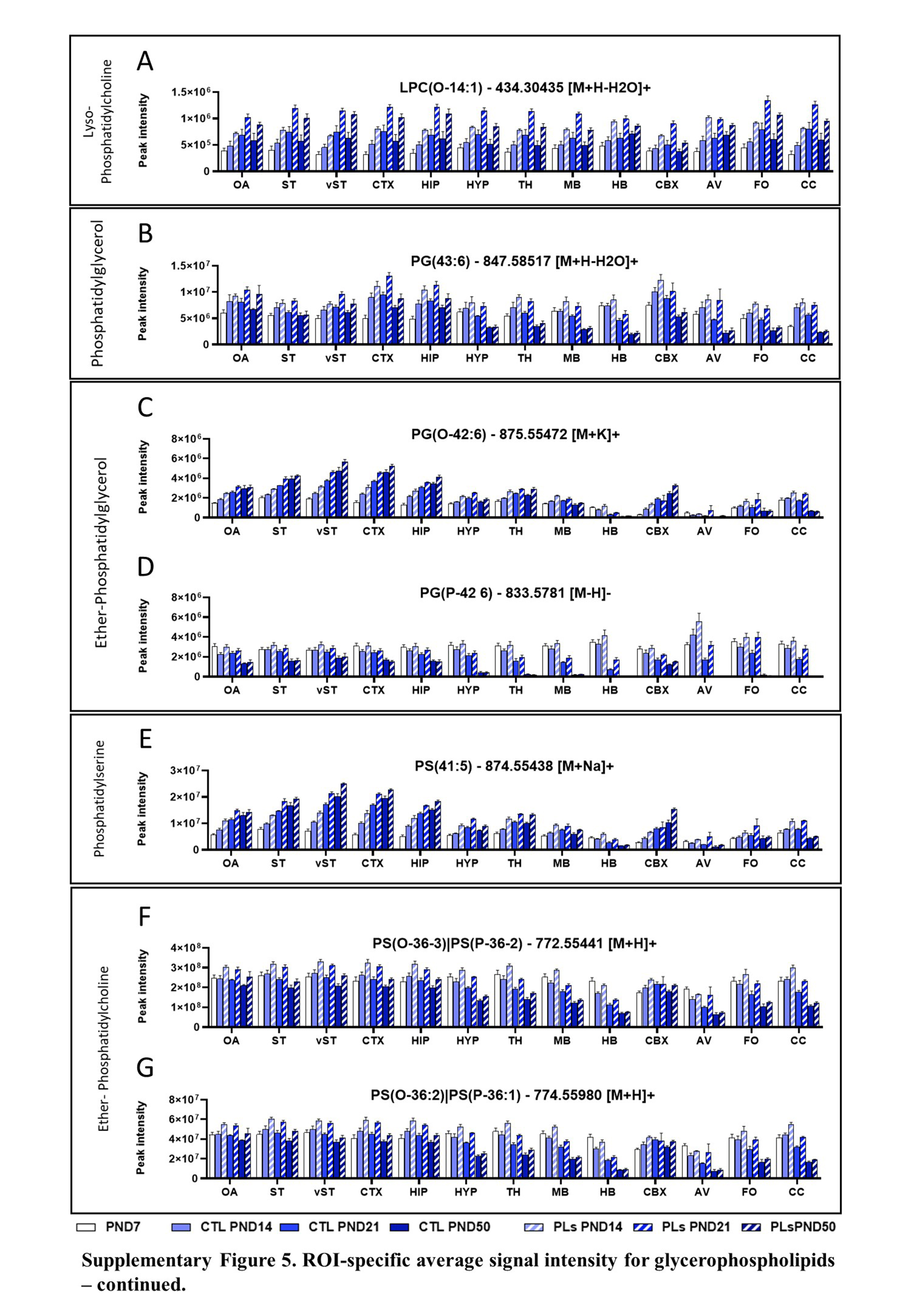

Supplement: Supplementary file 8 [file Image_5.JPEG]

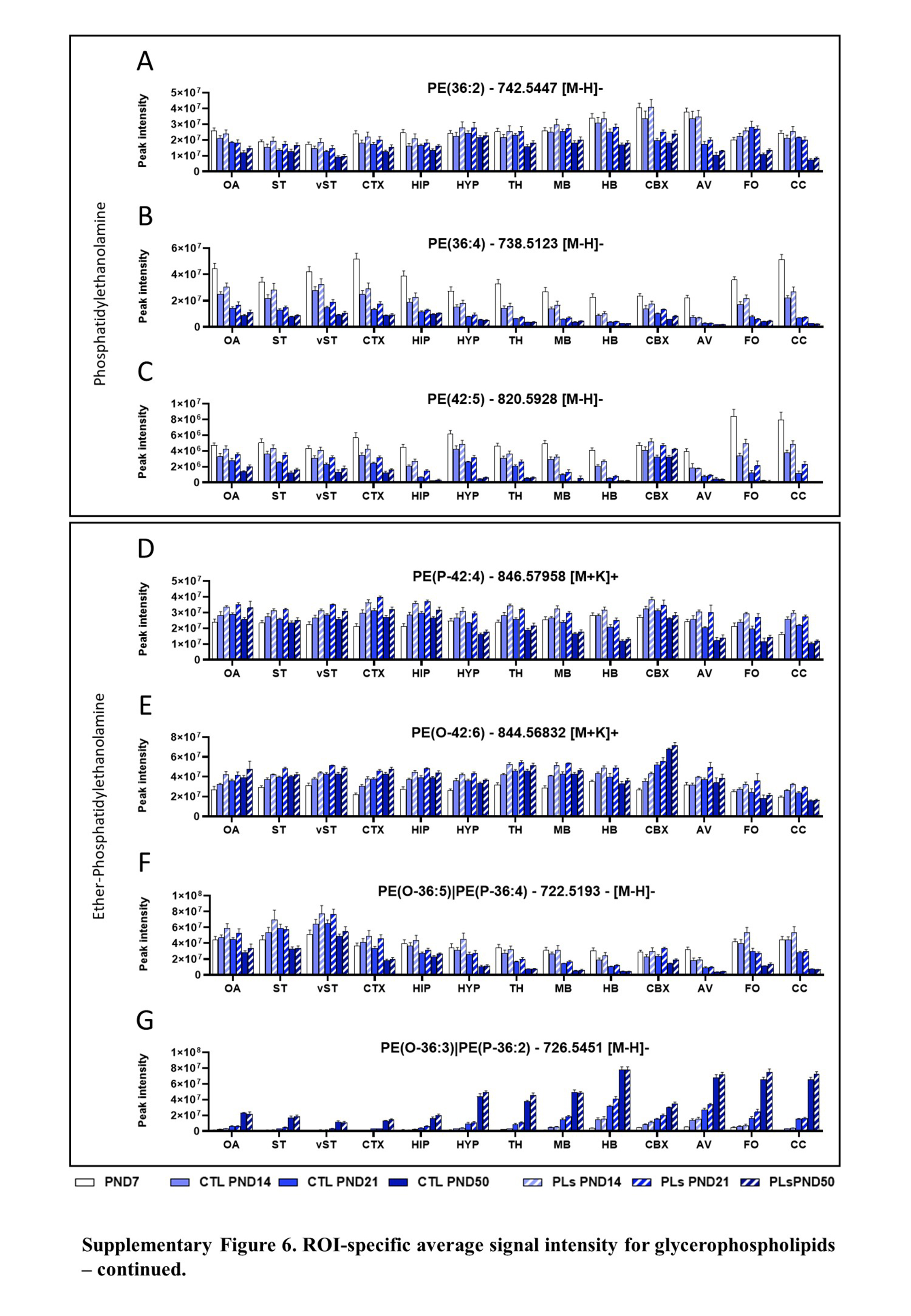

Supplement: Supplementary file 9 [file Image_6.JPEG]

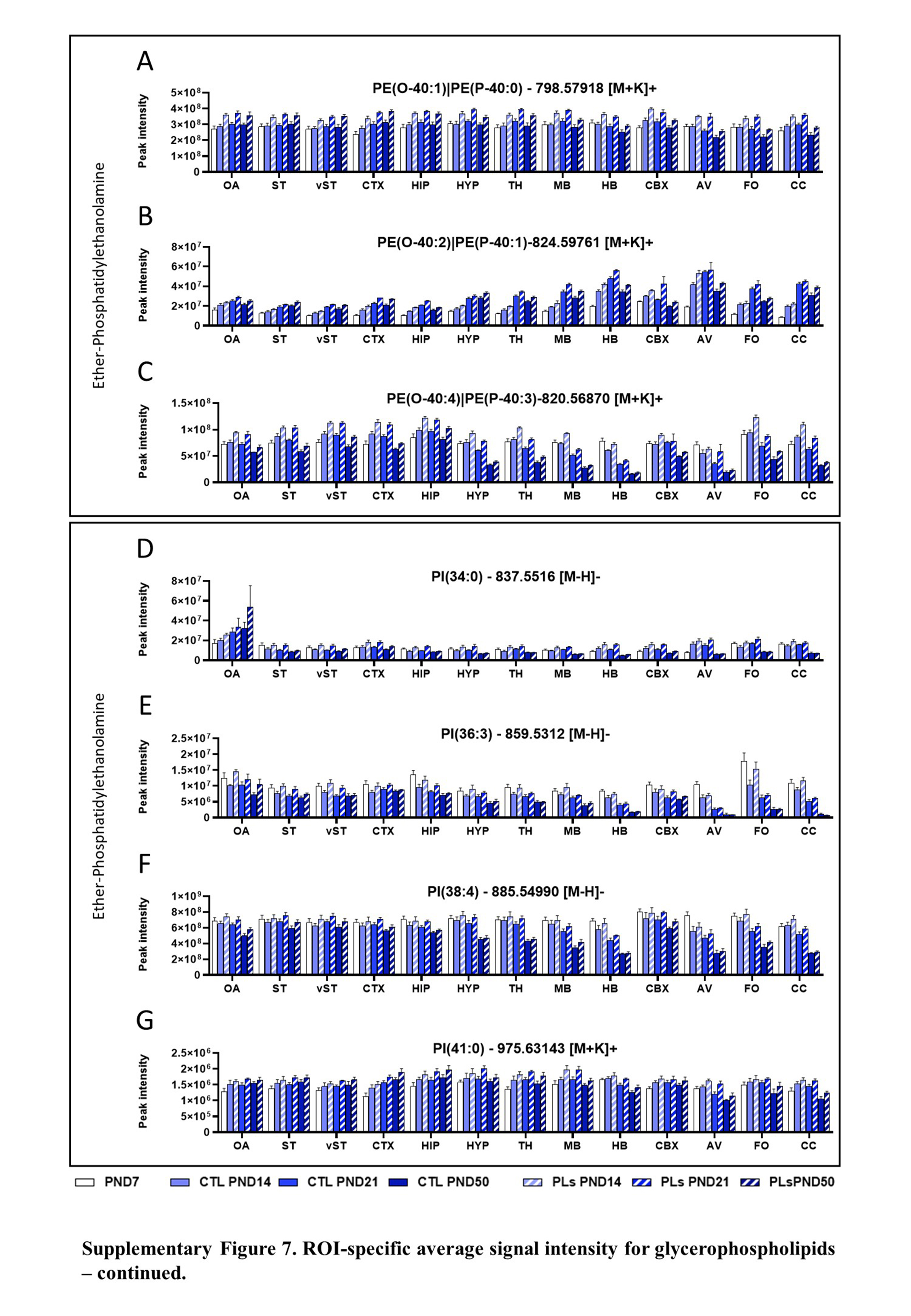

Supplement: Supplementary file 10 [file Image_7.JPEG]

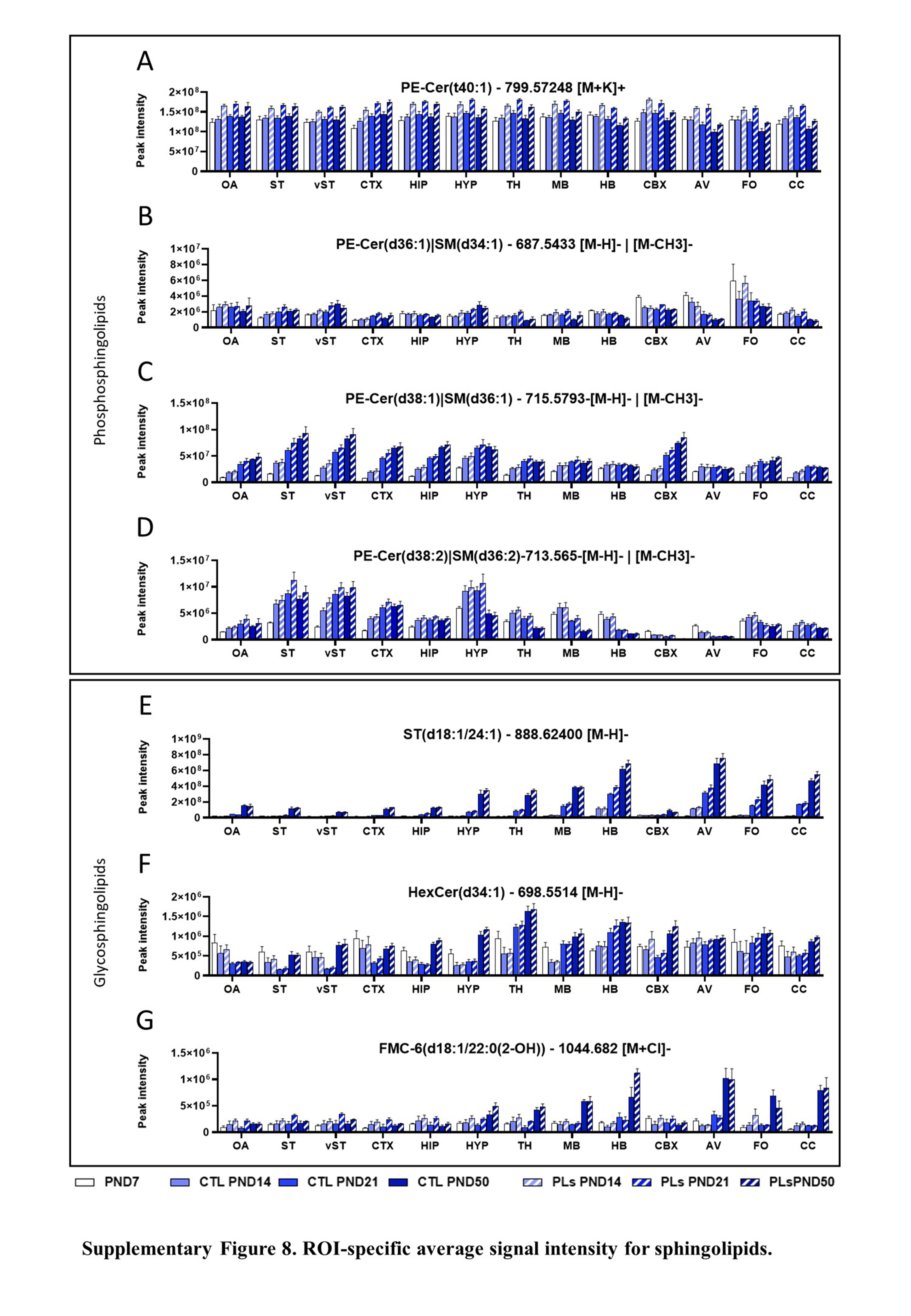

Supplement: Supplementary file 11 [file Image_8.JPEG]

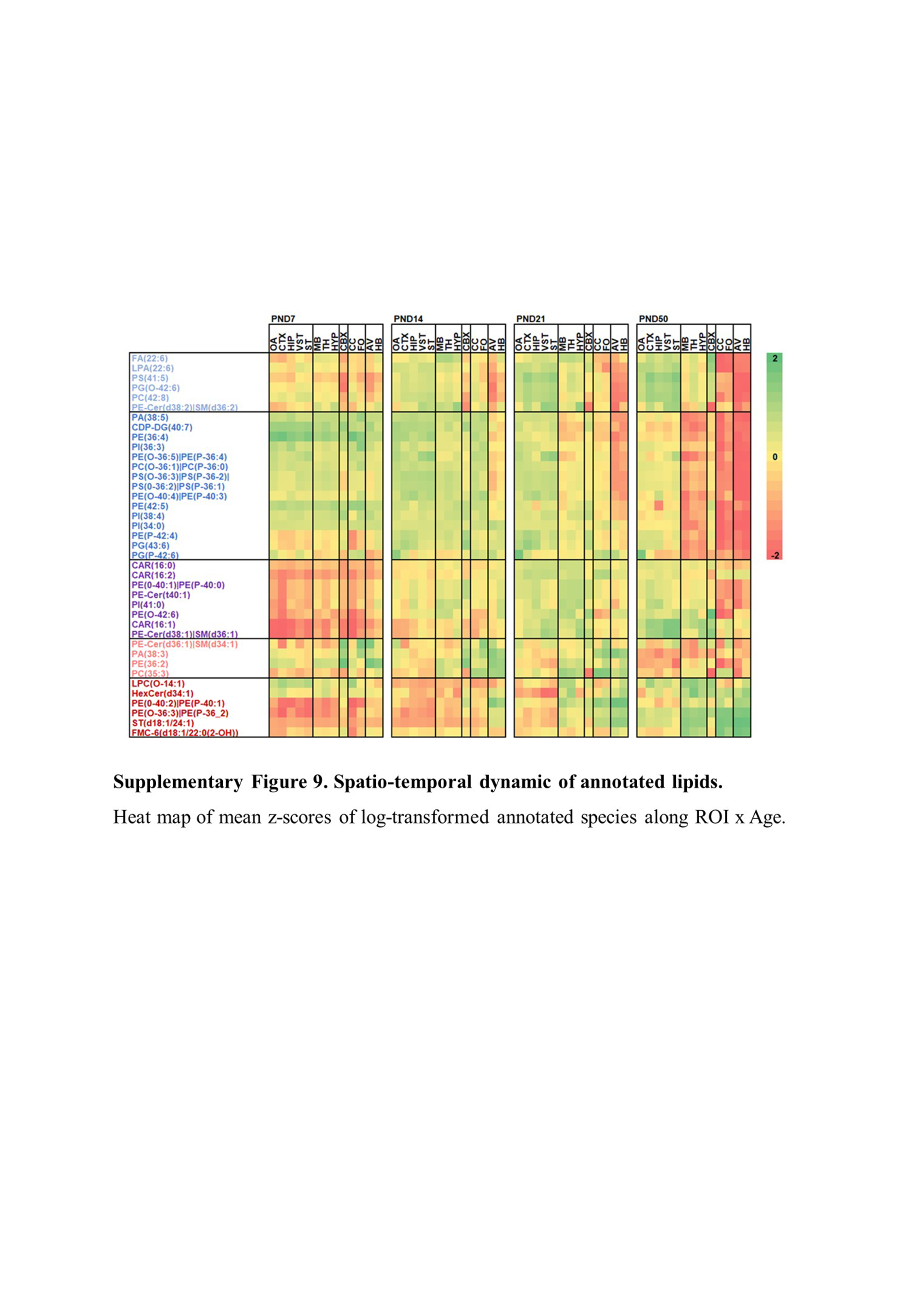

Supplement: Supplementary file 12 [file Image_9.jpg]
